# New insights into the protein aggregation pathology in myotilinopathy by combined proteomic and immunolocalization analyses

**DOI:** 10.1186/s40478-016-0280-0

**Published:** 2016-02-03

**Authors:** A. Maerkens, M. Olivé, A. Schreiner, S. Feldkirchner, J. Schessl, J. Uszkoreit, K. Barkovits, A. K. Güttsches, V. Theis, M. Eisenacher, M. Tegenthoff, L. G. Goldfarb, R. Schröder, B. Schoser, P. F. M. van der Ven, D. O. Fürst, M. Vorgerd, K. Marcus, R. A. Kley

**Affiliations:** Department of Neurology, Heimer Institute for Muscle Research, University Hospital Bergmannsheil, Ruhr-University Bochum, Buerkle-de-la-Camp-Platz 1, D-44789 Bochum, Germany; Medizinisches Proteom-Center, Ruhr-University Bochum, D-44801 Bochum, Germany; Department of Pathology and Neuromuscular Unit, Institute of Neuropathology, IDIBELL-Hospital Universitari de Bellvitge and Centro de Investigación Biomédica en Red de Enfermedades Neurodegenerativas (CIBERNED), Barcelona, Spain; Department of Neurology, Friedrich-Baur-Institute, Ludwig-Maximilians University of Munich, D-80336 Munich, Germany; Department of Cytology, Institute of Anatomy, Ruhr-University Bochum, D-44801 Bochum, Germany; Clinical Neurogenetics, National Institutes of Health, MSC 9404 Bethesda, MD USA; Institute of Neuropathology, University Hospital Erlangen, D-91054 Erlangen, Germany; Institute for Cell Biology, University of Bonn, D-53121 Bonn, Germany

**Keywords:** Myotilinopathy, Myofibrillar myopathy, Protein aggregation, Laser microdissection, Mass spectrometry, Immunolocalization study

## Abstract

**Introduction:**

Myofibrillar myopathies are characterized by progressive muscle weakness and impressive abnormal protein aggregation in muscle fibers. In about 10 % of patients, the disease is caused by mutations in the *MYOT* gene encoding myotilin. The aim of our study was to decipher the composition of protein deposits in myotilinopathy to get new information about aggregate pathology.

**Results:**

Skeletal muscle samples from 15 myotilinopathy patients were included in the study. Aggregate and control samples were collected from muscle sections by laser microdissection and subsequently analyzed by a highly sensitive proteomic approach that enables a relative protein quantification. In total 1002 different proteins were detected. Seventy-six proteins showed a significant over-representation in aggregate samples including 66 newly identified aggregate proteins. Z-disc-associated proteins were the most abundant aggregate components, followed by sarcolemmal and extracellular matrix proteins, proteins involved in protein quality control and degradation, and proteins with a function in actin dynamics or cytoskeletal transport. Forty over-represented proteins were evaluated by immunolocalization studies. These analyses validated our mass spectrometric data and revealed different regions of protein accumulation in abnormal muscle fibers. Comparison of data from our proteomic analysis in myotilinopathy with findings in other myofibrillar myopathy subtypes indicates a characteristic basic pattern of aggregate composition and resulted in identification of a highly sensitive and specific diagnostic marker for myotilinopathy.

**Conclusions:**

Our findings i) indicate that main protein components of aggregates belong to a network of interacting proteins, ii) provide new insights into the complex regulation of protein degradation in myotilinopathy that may be relevant for new treatment strategies, iii) imply a combination of a toxic gain-of-function leading to myotilin-positive protein aggregates and a loss-of-function caused by a shift in subcellular distribution with a deficiency of myotilin at Z-discs that impairs the integrity of myofibrils, and iv) demonstrate that proteomic analysis can be helpful in differential diagnosis of protein aggregate myopathies.

**Electronic supplementary material:**

The online version of this article (doi:10.1186/s40478-016-0280-0) contains supplementary material, which is available to authorized users.

## Introduction

Myofibrillar myopathies (MFM) comprise a genetically and clinically heterogeneous group of inherited muscle disorders characterized by focal disintegration of myofibrils predominantly at the Z-discs and by sarcoplasmic protein aggregation in muscle fibers [1–3]. In approximately 10 % of MFM patients, the disease is caused by mutations in the *MYOT* (synonym *TTID*) gene on chromosome 5q31 [4, 5]. This gene encodes the 57 kDa Z-disc protein myotilin [6], an actin-cross-linking protein that plays an important role in sarcomere assembly and stabilization of myofibrillar Z-discs [6, 7]. Myotilin consists of a unique serine-rich amino terminal region, two Ig-like domains necessary for the formation of antiparallel myotilin dimers, and a short carboxy terminal tail [6, 7]. It directly binds filamin C [8], α-actinin [6, 7], FATZ-1 [9] and ZASP [10].

MFM associated with myotilin mutations, hereafter referred to as myotilinopathy, usually manifests between the 5th and 8th decade of life with progressive muscle weakness most often starting at distal lower limbs [4, 5, 11]. Peripheral neuropathy, cardiomyopathy and respiratory failure have been described in a subset of patients but are not constant features of myotilinopathy [4, 11, 12].

Histological characteristics of myotilinopathy include variation of fiber size, fibro-fatty tissue proliferation, type I fiber predominance, core-like lesions, rimmed vacuoles and non-rimmed vacuolated areas. Indeed, the most prominent myopathological features in modified trichrome stain are pleomorphic protein deposits in so-called abnormal fibers described as hyaline dense inclusions, non-hyaline amorphous inclusions, spheroid inclusion bodies or nemaline-like bodies [11, 13]. Immunohistochemical studies identified an accumulation of several proteins in these deposits, including myotilin and other Z-disc proteins, ectopically expressed sarcolemmal proteins and proteins involved in protein degradation pathways [4, 5, 11, 14–16]. However, since these immunolocalization studies were hypothesis-driven and restricted to pre-selected proteins, the precise constitution of deposits and the mechanisms of protein aggregation are largely unknown.

We established a highly sensitive proteomic approach to decipher the composition of MFM aggregates selectively collected from abnormal fibers by laser microdissection [17, 18]. We already applied this method in filaminopathy and desminopathy [17, 18], two MFM subtypes caused by mutations in *FLNC* and *DES*, respectively. Analysis of samples from six filaminopathy [17] and five desminopathy [18] patients revealed several novel aggregate components and provided new insights into the pathomechanisms of these diseases. In addition, subtype specific proteomic profiles were detected that can be helpful in differential diagnosis of protein aggregate myopathies.

We here present a proteomic study in myotilinopathy, including samples from 15 patients harboring four different *MYOT* mutations. We used our established combined laser microdissection and mass spectrometry approach to identify new disease-relevant proteins that accumulate in abnormal fibers. Extensive immunofluorescence studies were performed to validate our proteomic findings and to get a deeper insight into the subcellular distribution of proteins. Furthermore, we compared the results with our previous findings in filaminopathy and desminopathy in order to identify specific proteomic markers for myotilinopathy.

## Materials and methods

### Patients

Skeletal muscle samples from 15 myotilinopathy patients with a histological phenotype typical of MFM and with known pathogenic mutations in exon 2 of *MYOT* were included in this study. Two patients carried a p.Lys36Glu mutation [11], one patient a p.Ser55Phe mutation [4, 11], six patients a p.Ser60Cys mutation [4, 11], and six patients a p.Ser60Phe mutation [4, 11]. More detailed data of patients and muscle samples are presented in Table 1.

**Table 1:** Overview of myotilinopathy patients included in this study

ID	Gender	Age at biopsy [years]	Muscle	*MYOT* mutation
1	Male	78	biceps brachii	p.Ser60Phe
2	Female	64	gastrocnemius	p.Ser60Cys
3	Female	74	vastus lateralis	p.Ser55Phe
4	Female	82	tibialis anterior	p.Ser60Phe
5	Male	80	vastus lateralis	p.Lys36Glu
6	Female	81	vastus lateralis	p.Lys36Glu
7	Male	78	biceps brachii	p.Ser60Phe
8	Male	52	vastus lateralis	p.Ser60Cys
9	Male	72	gastrocnemicus	p.Ser60Cys
10	Male	77	tibialis anterior	p.Ser60Cys
11	Female	85	vastus lateralis	p.Ser60Cys
12	Male	65	vastus lateralis	p.Ser60Phe
13	Female	69	deltoideus	p.Ser60Cys
14	Male	71	tibialis anterior	p.Ser60Phe
15	Male	80	vastus lateralis	p.Ser60Phe

### Proteomic analysis

**Fig. 1:**
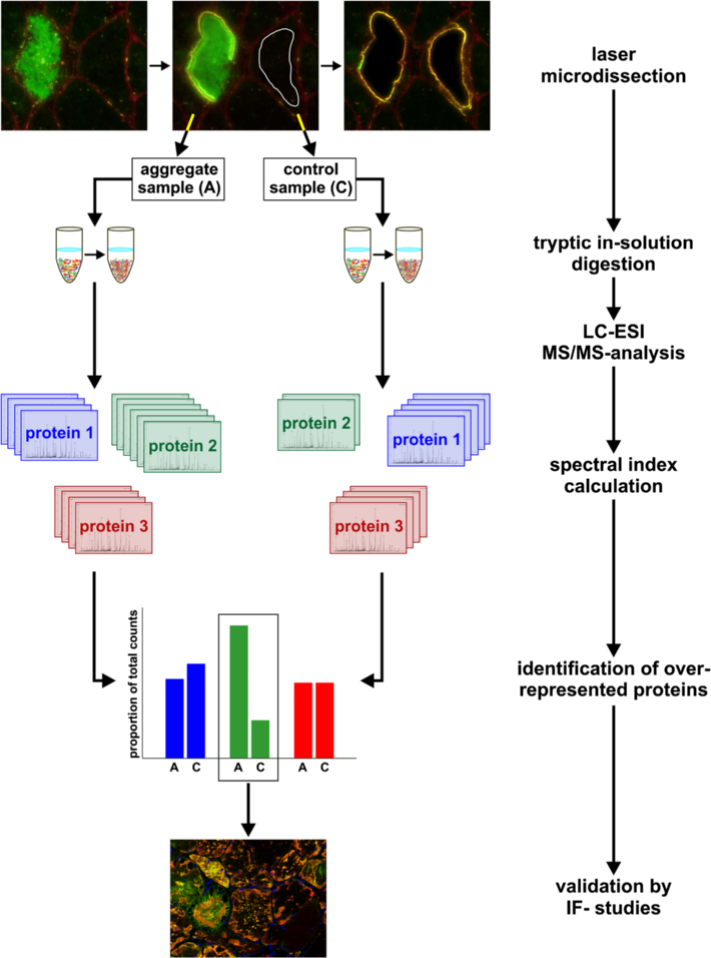
Workflow of our combined laser microdissection and label-free proteomic approach. Frozen skeletal muscle sections from myotilinopathy patients were immunostained using a primary antibody directed against myotilin in order to detect protein aggregation in affected muscle fibers. Protein aggregates (“aggregate samples”) and aggregate-free areas in fibers with a normal appearance (“control samples”) were collected from the same section by laser microdissection (LMD). After tryptic in-solution digestion of extracted proteins, protein identification was performed by LC-ESI-MS/MS-analysis. Proteins with an over-representation in aggregate samples compared to control samples were identified by label-free relative quantification (spectral index calculation). For validation of proteomic data, serial cryosections of muscle samples from myotilinopathy patients were immunostained with primary antibodies directed against selected proteins identified as over-represented in aggregate samples

A combined laser microdissection and label free mass spectrometry approach (see workflow in Fig. 1) was applied as described by us before [17, 18] with modifications.

#### Laser Microdissection and sample processing

Frozen sections (10 μm) of skeletal muscle samples (see above) were used for sample collection by laser microdissection. Immunofluorescence staining with a primary antibody directed against myotilin (Additional file 1: Table S1) to detect protein aggregation in abnormal muscle fibers was performed as described [18]. A total area of 250,000 μm^2^ of protein aggregates and surrounding sarcoplasm (hereinafter referred as “aggregate samples”) and the same area of sarcoplasm of aggregate-free muscle fibers from the same sections (intraindividual “control samples”) were collected into tubes with 40 μl formic acid (FA, 98–100 %) by laser microdissection (LMD 6500, Leica Microsystems, Wetzlar, Germany). After incubation for 30 min at room temperature and sonification (35 kHz) for 5 min (RK31, BANDELIN electronic, Berlin, Germany) the samples were centrifuged for 10 min (12,000 g, 4 °C) and frozen at −80 °C. Subsequently, a tryptic in solution-digestion was performed as described [18].

#### Mass spectrometry and relative protein quantification

The samples were analyzed by nanoHPLC-ESI-MS/MS-analysis. Nano-HPLC was performed on an UltiMate 3000 RSLC nano LC system (Thermo Fisher Scientific, Dreieich, Germany). Samples were loaded on a trap column (Acclaim®PepMap 100, 100 μm × 2 cm, C18, particle size 5 μm, pore size 100 Å) with 0.1 % TFA (flow rate 10 μl/min). After sample concentration and a washing step the trap column was serially connected with an analytical column (Acclaim®PepMap RSLC, 75 μm x 50 cm, C18, particle size 2 μm, pore size 100 Å), and peptides were separated with a flow rate of 400 nl/min by using a solvent gradient from 4 % A to 40 % B (A: 0.1 % FA, B: 84 % acetonitrile, 0.1 % FA) for 95 min. The HPLC system was directly coupled to a nanoelectrospray ionization source of a LTQ Orbitrap Elite mass spectrometer (Thermo Fisher Scientific, Dreieich, Germany). Ionization was performed using a nanoelectrospray source with a spray voltage of 1500 V and capillary temperature of 275 °C. The mass spectrometer was operated in a data dependent acquisition. Full MS spectra were scanned between 300 and 2000 m/z (resolution 60,000, AGC 1 × 10^6^) to detect precursor ions. Lock mass polydimethylcyclosiloxane (m/z 445.120) was used for internal calibration. The m/z values initiating MS/MS were set on a dynamic exclusion list for 35 s and the 20 most intensive ions (charge >1) were selected for MS/MS-fragmentation. Fragments were generated by low-energy collision-induced dissociation with normalized collision energy of 35 % (isolation width 2, activation time 10 ms). After ESI-MS/MS analysis mass spectrometric data were searched against a human protein database containing the entire Uniprot/Swissprot (release 2014/10, 546,790 entries) using Mascot search engine (Matrixscience, London, UK) with the following search parameters: peptide mass tolerance 10 ppm, fragment mass tolerance 0.5 Da, one missed cleavage and carbamidomethylation (C), oxidation (M), phosphorylation (S, T, Y) as variable modifications. The quality of identified peptide spectrum matches was evaluated by calculating a FDR threshold of 1 % using the in-house developed software “PIA-Protein Inference Algorithm” [19] (http://mpc-bioinformatics.github.io/pia/). Only peptides that were unique within the complete data set were considered for final analysis to prevent an assignment to different proteins due to sequence homologies. Protein quantification was performed by summing up all peptide spectrum matches (hereinafter referred as “spectral counts”) belonging to the respective protein followed by a normalization step. For normalization the proportion (in ‰) of spectral counts for each protein based on total sum of spectral counts in the individual samples was calculated. To identify proteins that were over-represented in aggregate samples, the ratios between the averaged proportions in aggregate and control samples were calculated and a two-tailed unpaired t-test (equal variances assumed) was conducted for each protein. A protein was considered as significantly over-represented when the aggregate to control ratio was >1.5 and the *p*-value <0.05. In addition, we compared the results of proteomic analysis in myotilinopathy patients with data from our previous studies in filaminopathy and desminopathy [17, 18] using the same modified parameters described above. For identification of diagnostic markers for myotilinopathy, proteomic data from 10 myotilinopathy patients (ID 1–10) were evaluated for differences in ratios and proportions of proteins compared to findings in filaminopathy and desminopathy. Potential markers were subsequently checked in the remaining 5 myotilinopathy patients (ID 11–15) in a prospective manner. Specificity and sensitivity were calculated using 2 × 2 contingency tables.

### Validation of proteomic data by immunolocalization studies

We used double immunofluorescence staining to assess the localization of selected proteins that were identified as over-represented in aggregate samples by our proteomic analysis. 5-μm-thick serial frozen skeletal muscle sections from three myotilinopathy patients (IDs 1, 10 and 11) were incubated overnight at 4 °C with primary antibodies directed against 40 different proteins (Additional file 1: Table S1). Myotilin or filamin C immunostaining was used to identify abnormal fibers with protein deposits. After three washing steps with PBS for 5 min the sections were incubated with isotype specific secondary antibodies conjugated with DyLight 488 (Dianova, Hamburg, Germany; dilution 1:1000) or Texas Red (Jackson Immuno Research, West Grove, Pennsylvania, USA; dilution 1:500) for 45 min at RT, followed by three washing steps with PBS for 5 min. Nuclei were displayed by incubation with 4’, 6-diamidino-2-phenylindole (DAPI) (Roche Diagnostics, Indianapolis, IN, USA; dilution 1:10,000) for 1 min at 37 °C. The staining procedure was finished by two washing steps with PBS for 5 min. Stained sections were mounted in Roti®-Mount FluorCare (Carl Roth, Karlsruhe, Germany) and analyzed using an IX83 inverted microscope system (Olympus, Hamburg, Germany).

## Results

### Proteomic analysis in myotilinopathy

Mass spectrometric analysis of aggregate and control samples identified 1002 different proteins. 72 of these proteins showed a statistically significant over-representation in aggregate samples with a ratio >1.5 compared to control samples (Table 2, for UniProtKB/Swiss-Prot accession numbers and gene names see Additional file 2: Table S2). Although the last four proteins in Table 2 were detected as over-represented in aggregate areas with their individual p values (between 0.05 and 0.08) slightly below the level of statistical significance, these proteins were also evaluated by immunofluorescence studies. The results confirmed an accumulation in abnormal muscle fibers (see below).

**Table 2:** Proteins identified as over-represented in aggregate samples in myotilinopathy

	Mean proportion [‰]^a^			
**Protein**	Aggregates	Control	Ratio	*p* value
**Desmin**	52.09	14.36	3.6	6.75 × 10^−6^
**Filamin C**	31.82	17.86	1.8	6.69x10^−6^
**Myotilin**	24.05	4.51	5.3	2.91 × 10^−8^
**Xin actin-binding repeat-containing protein 2 (XIRP2)**	21.75	0.10	214.8	7.66 × 10^−6^
**Nebulin-related-anchoring protein (N-RAP)**	18.19	0.57	31.7	7.31 × 10^−7^
**Plectin**	14.27	6.75	2.1	0.0003
**Collagen alpha-3(VI) chain**	11.22	1.99	5.6	1.88 × 10^−5^
**Obscurin**	8.49	4.68	1.8	0.0020
**Xin actin-binding repeat-containing protein 1 (Xin)**	7.17	0.67	10.7	1.88 × 10^−7^
**Alpha-crystallin B chain (αB-crystallin)**	6.50	1.08	6.0	1.03 × 10^−5^
**Nestin**	5.14	0.11	47.3	7.62 × 10^−6^
Myosin-3	4.16	1.13	3.7	0.0043
Histone H4	3.87	1.23	3.1	3.08 × 10^−5^
**Collagen alpha-1(VI) chain**	3.73	0.80	4.7	0.0005
**Myosin-binding protein H (MYBPH)**	3.19	0.51	6.3	0.0029
Fibrillin-1	2.90	0.58	5.0	0.0230
**Dysferlin**	2.71	0.33	8.2	2.91 × 10^−6^
**Prelamin-A/C**	2.67	0.56	4.8	2.44 × 10^−5^
PDZ and LIM domain protein 3	2.56	1.26	2.0	0.0029
**Collagen alpha-2(VI) chain**	2.41	0.52	4.6	0.0004
**Heat shock cognate 71 kDa protein (HSPA8/Hsc70)**	2.40	0.99	2.4	0.0001
Collagen alpha-1(I) chain	2.05	0.53	3.8	0.0475
**Sequestosome-1 (p62)**	1.75	0.03	52.0	0.0001
Collagen alpha-2(I) chain	1.62	0.58	2.8	0.0080
**Tubulin alpha-4A chain**	1.51	0.79	1.9	0.0074
**Heat shock protein beta-1 (HSPB1/Hsp27)**	1.40	0.44	3.2	0.0173
**Basement membrane-specific heparan sulfate proteoglycan core protein (Perlecan)**	1.25	0.26	4.8	0.0077
**Laminin subunit gamma-1**	1.25	0.06	20.6	0.0001
NADH-cytochrome b5 reductase 1	1.13	0.69	1.6	0.0385
Laminin subunit beta-2	1.07	0.23	4.6	0.0110
Kelch-like protein 40	1.07	0.49	2.2	0.0207
**Dystrophin**	1.04	0.12	8.6	0.0004
Collagen alpha-2(IV) chain	0.97	0.31	3.1	0.0016
**Heat shock 70 kDa protein 1A/1B**	0.90	0.42	2.2	0.0030
ATP synthase F(0) complex subunit B1, mitochondrial	0.90	0.48	1.9	0.0121
Tubulin beta chain	0.79	0.29	2.8	0.0016
**Decorin**	0.79	0.08	9.7	0.0230
**BAG family molecular chaperone regulator 3 (BAG3)**	0.71	0.14	5.2	0.0007
Collagen alpha-1(III) chain	0.69	0.19	3.6	0.0348
**Microsomal glutathione S-transferase 3 (MGST3)**	0.68	0.27	2.5	0.0365
Synaptophysin-like protein 2	0.57	0.19	2.9	0.0087
Myosin-14	0.52	n.d.	n.a.	0.0203
**Muscle-related coiled-coil protein (MURC)**	0.49	n.d.	n.a.	0.0491
NADH-cytochrome b5 reductase 3	0.45	0.07	6.8	0.0010
**Myopalladin**	0.44	n.d.	n.a.	0.0027
**Microtubule-associated protein 4 (MAP4)**	0.43	0.02	25.9	0.0110
Sarcolemmal membrane-associated protein	0.40	0.10	4.0	0.0131
**Protein kinase C and casein kinase substrate in neurons protein 3 (PACSIN 3)**	0.40	0.02	20.2	0.0041
**Next to BRCA1 gene 1 protein (NBR1)**	0.37	n.d.	n.a.	0.0057
**Supervillin**	0.31	0.09	3.3	0.0315
**14-3-3 protein gamma**	0.30	0.08	3.8	0.0134
**Tropomodulin-1**	0.29	0.02	15.0	0.0019
**Syncoilin**	0.28	n.d.	n.a.	0.0021
Voltage-dependent calcium channel subunit alpha-2/delta-1	0.28	0.02	16.7	0.0084
Histone H1.0	0.26	n.d.	n.a.	0.0016
Signal recognition particle subunit SRP68	0.26	0.04	6.0	0.0258
Unconventional myosin-XVIIIa	0.26	n.d.	n.a.	0.0043
Tubulin beta-4B chain	0.24	0.04	5.8	0.0013
Xaa-Pro aminopeptidase 1	0.23	n.d.	n.a.	0.0018
Myeloid leukemia factor 2	0.22	n.d.	n.a.	0.0021
Myosin-10	0.17	n.d.	n.a.	0.0455
Elastin	0.16	n.d.	n.a.	0.0465
Protein NipSnap homolog 2	0.16	n.d.	n.a.	0.0254
60S ribosomal protein L12	0.16	0.03	6.3	0.0355
**Heat shock protein beta-8 (HSPB8/Hsp22)**	0.15	n.d.	n.a.	0.0053
Epoxide hydrolase 1	0.15	n.d.	n.a.	0.0461
**Y-box-binding protein 3 (YBX3)**	0.12	n.d.	n.a.	0.0141
60S ribosomal protein L22	0.12	n.d.	n.a.	0.0490
60S ribosomal protein L7	0.10	n.d.	n.a.	0.0330
Collagen alpha-3(IV) chain	0.09	n.d.	n.a.	0.0337
CD59 glycoprotein	0.09	n.d.	n.a.	0.0333
SET-binding protein	0.09	n.d.	n.a.	0.0333
**78 kDa glucose-regulated protein (GRP78/BiP)**	0.29	0.05	5.7	0.0526^*^
**Cysteine and glycine-rich protein 3 (CSRP3)**	0.21	0.08	2.8	0.0504^*^
**Delta-sarcoglycan**	0.07	n.d.	n.a.	0.0781^*^
**Thrombospondin-4**	0.07	n.d.	n.a.	0.0727^*^

*n.d*. not detected, *n.a*. not applicable. Protein names marked in bold letters: proteins included in immunolocalization studies

^*^ *p* value between 0.05 and 0.1 but validated by immunofluorescence studies

^a^per mill of total spectral counts

**Fig. 2:**
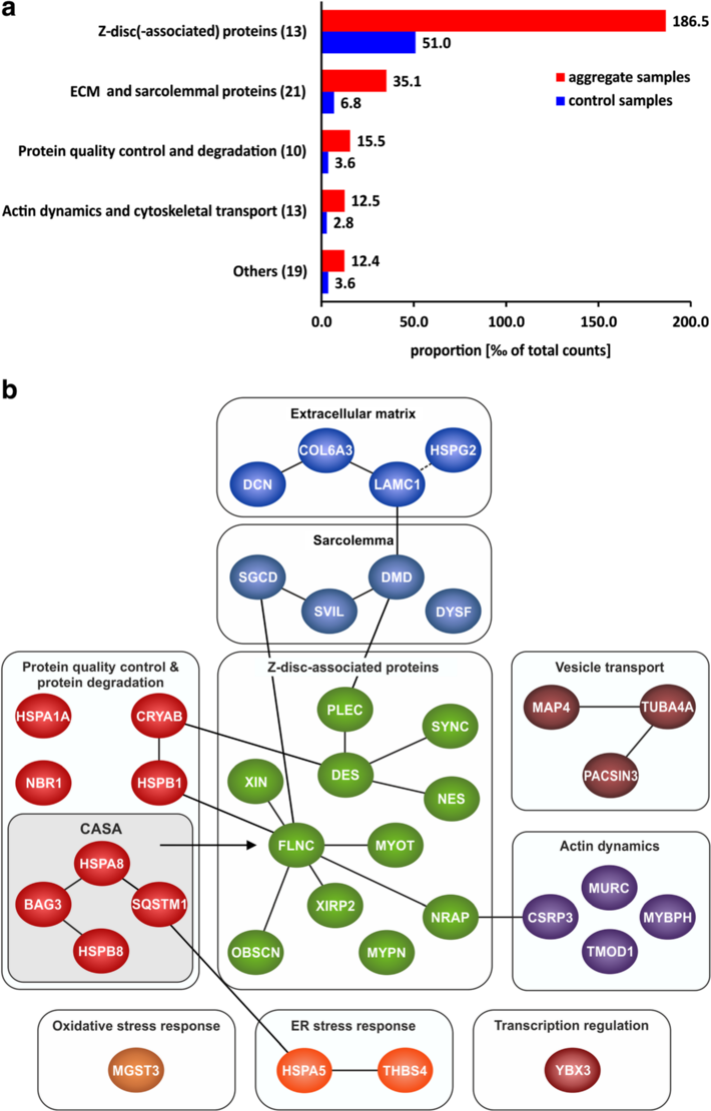
Functional classification and interactions of proteins identified as over-represented in myotilinopathy aggregates samples. **a** Results of proteomic analysis in myotilinopathy. Proteins identified as over-represented in aggregate samples by mass spectrometric analysis are grouped with regard to their localization or main function. Shown are proportions in aggregate and control samples. Z-disc (−associated) proteins constituted the most abundant group of over-represented proteins, followed by proteins of the sarcolemma and extracellular matrix (ECM), proteins involved in protein quality control and degradation and proteins with a function in actin dynamics or cytoskeletal transport. **b** Schematic illustration of over-represented proteins which were verified by immunofluorescence studies and of protein-protein interactions. Each protein is termed by the name of its encoding gene (see Additional file 2: Table S2). Direct protein interactions, identified by review of the literature, are depicted by solid connecting lines. Indirect protein interactions are depicted by a dashed connecting line

A functional classification of proteins by review of the literature revealed that Z-disc and Z-disc-associated proteins, including the disease causing protein myotilin, were most abundantly over-represented in aggregate samples, followed by sarcolemmal and extracellular matrix proteins, proteins involved in protein quality control and degradation, and proteins with a function in actin dynamics or cytoskeletal transport (Fig. 2). Proteomic profiles of aggregates collected from different muscles were largely homogeneous and independent of the specific *MYOT* mutation (Additional file 3: Figure S1).

### Comparison of mass spectrometric analysis in different MFM subtypes

**Fig. 3:**
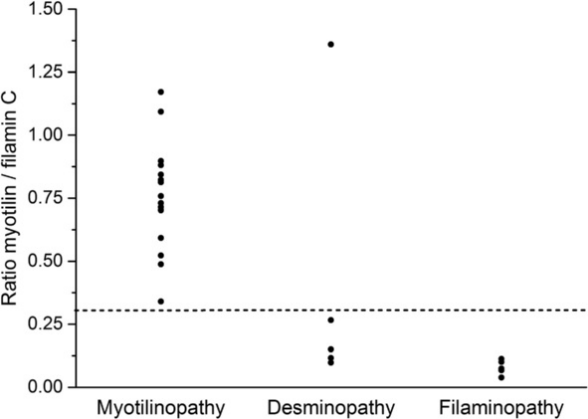
Individual myotilin/filamin C ratios in aggregate samples from myotilinopathy, desminopathy and filaminopathy patients. Proportions of myotilin and filamin C in aggregate samples were determined by our proteomic approach. The myotilin/filamin C ratio was always above 0.3 in samples from 15 myotilinopathy patients and below 0.3 in all filaminopathy cases (*n* = 6) and in 4 out of 5 desminopathy patients

The comparison of data from proteomic analysis in myotilinopathy, filaminopathy and desminopathy revealed that desmin, filamin C, myotilin, N-RAP, Xin, Xirp2, αB-crystallin, and nestin were always over-represented in aggregate samples irrespective of the MFM subtype, although differences in ratios and proportions were detected. The ratio of the myotilin and filamin C proportions in aggregate samples was identified as a new diagnostic marker for myotilinopathy with a high sensitivity (100 %) and specificity (91 %) in our MFM cohort (Fig. 3). This ratio was always above 0.3 in samples from myotilinopathy patients (range 0.34–1.17) and below 0.3 in all filaminopathy (range 0.07–0.1) and in 4 out of 5 desminopathy patients (range 0.11–0.26).

Our previous proteomic studies [18] identified the filamin C ratio (proportion in aggregate samples divided by proportion in control samples) as a diagnostic marker to differentiate filaminopathy from desminopathy. This ratio was always above 6 in filaminopathy samples (range 6.6–8.6) and below 5 in desminopathy samples (range 1.9–4.5). Our data presented here revealed an intraindividual filamin C ratio below 4 (range 1.1–3.9) in all 15 myotilinopathy patients indicating that the filamin C ratio is also suited for differentiation of filaminopathy from myotilinopathy (sensitivity of this diagnostic marker to detect filaminopathy in our MFM cohort 100 %, specificity 100 %).

### Validation of proteomic data by immunofluorescence studies

Our extensive immunolocalization studies confirmed the results of proteomic analysis. Forty proteins detected as over-represented in aggregate samples also showed increased immunoreactivities in fibers harboring protein aggregates. However, the distribution pattern of protein accumulation varied considerably between individual proteins.

**Fig. 4:**
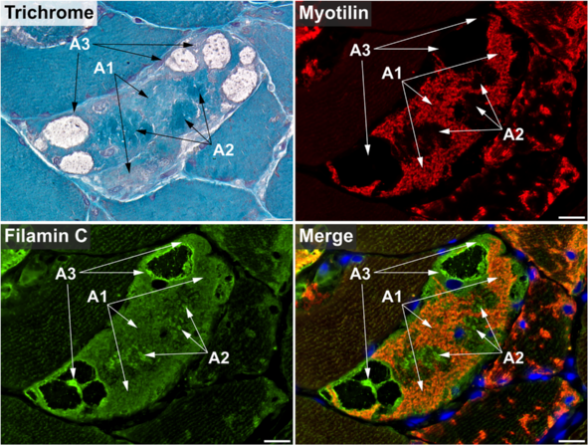
Abnormal areas in skeletal muscle fibers with protein aggregates in myotilinopathy. Serial skeletal muscle cryosections from a myotilinopathy patient (ID1 in Table 1) were stained with modified Gomori trichrome and double-immunostained with primary antibodies directed against myotilin and filamin C. DAPI was used to visualize nuclei. Three different areas of protein accumulation were detected in abnormal fibers: Abnormal areas A1 appear hyaline on trichrome stain and are characterized by aggregation of myotilin and several other proteins (see Table 3) including filamin C. Abnormal areas A2 are located in the sarcoplasm of abnormal fibers, appear dark blue in trichrome stain and are characterized by a decreased immunoreactivity for myotilin and a strong accumulation of filamin C (among other proteins, see Table 3). Abnormal areas 3 are located around subsarcolemmal vacuoles in abnormal fibers, appear hyaline-purple in trichrome stain and show a decreased immunoreactivity for myotilin and an increased immunoreactivity for filamin C (and other proteins, see Table 3). Scale bar = 20 μm

**Fig. 5:**
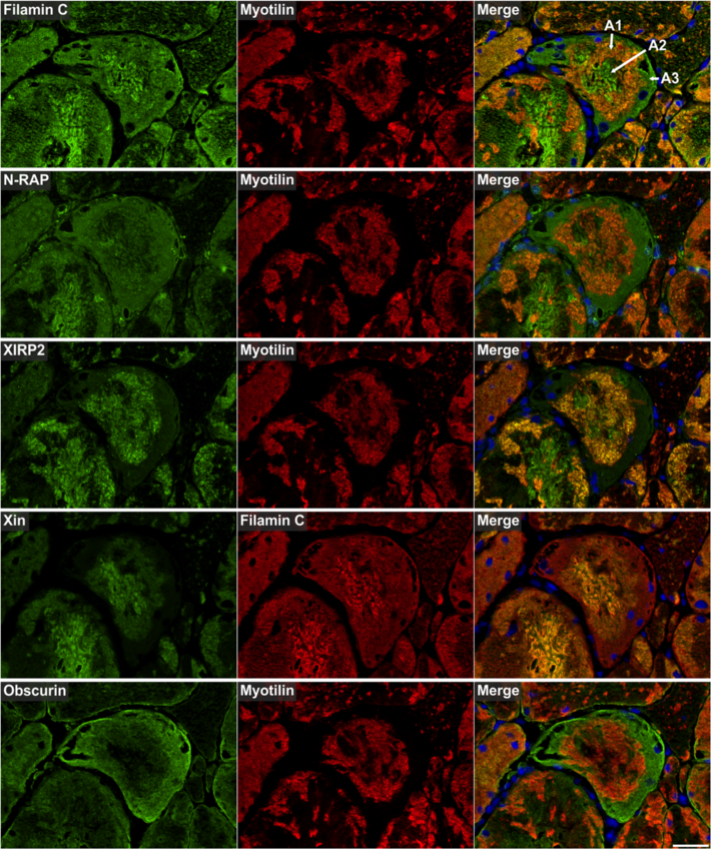
Immunolocalization of Z-disc (−associated) proteins I. Serial skeletal muscle cryosections from a myotilinopathy patient (ID1 in Table 1) were double-stained with antibodies recognizing the indicated Z-disc (−associated) proteins and either filamin C or myotilin as a positive control to localize abnormal fibers. Nuclei were visualized by DAPI staining. Results of semiquantitative evaluation of signal intensities in the different abnormal areas A1–A3 are specified in Table 3. A1: abnormal area 1; A2: abnormal area 2; A3: abnormal area 3. Scale bar = 50 μm

**Fig. 6:**
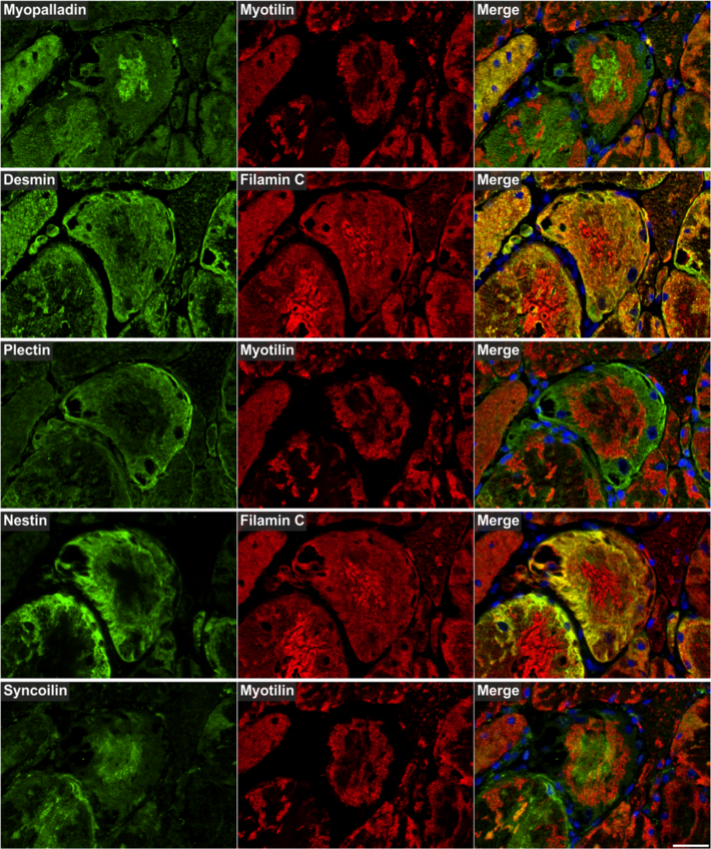
Immunolocalization of Z-disc (−associated) proteins II. Double immunofluorescence staining was performed on serial skeletal muscle cryosections from a myotilinopathy patient (ID1 in Table 1) using antibodies directed against myopalladin, desmin, plectin, nestin, and syncoilin and either filamin C or myotilin. Nuclei were visualized by the blue-fluorescent DAPI nucleic acid stain. Data of semiquantitative assessment of immunofluorescence intensities in abnormal areas A1–A3 are listed in Table 3. Scale bar = 50 μm

**Fig. 7:**
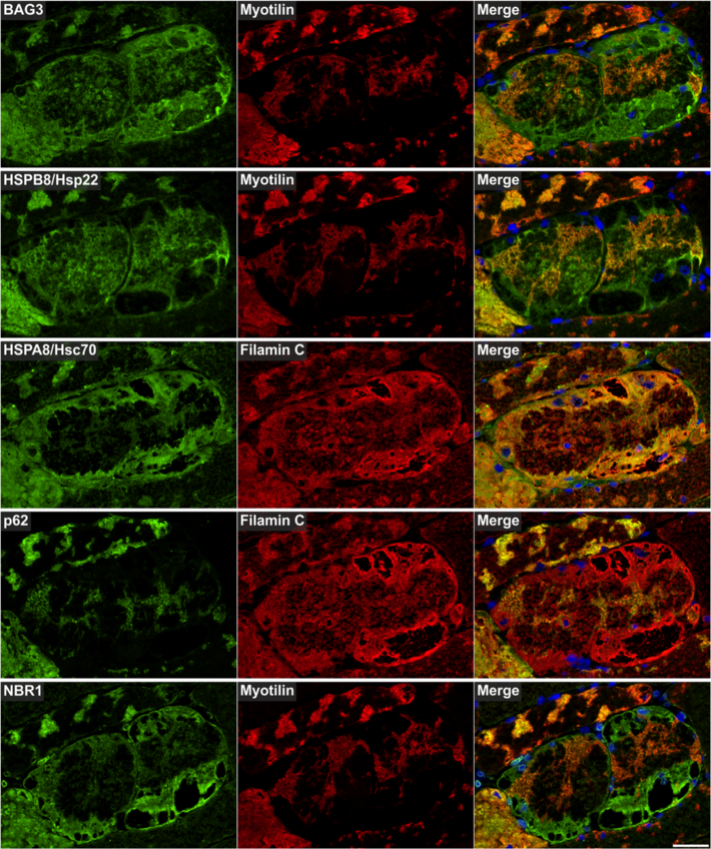
Immunolocalization of proteins involved in chaperone-assisted selective autophagy (CASA) and NBR1-mediated selective autophagy. Serial skeletal muscle cryosections from a myotilinopathy patient (ID1 in Table 1) were double-stained with antibodies recognizing the indicated CASA components (BAG3, HSPB8/Hsp22, HSPA8/Hsc70, p62) and NBR1 and either filamin C or myotilin. Nuclei were visualized by DAPI staining. Results of semiquantitative evaluation of signal intensities in the different abnormal areas A1–A3 are specified in Table 3. Scale bar = 50 μm

**Fig. 8:**
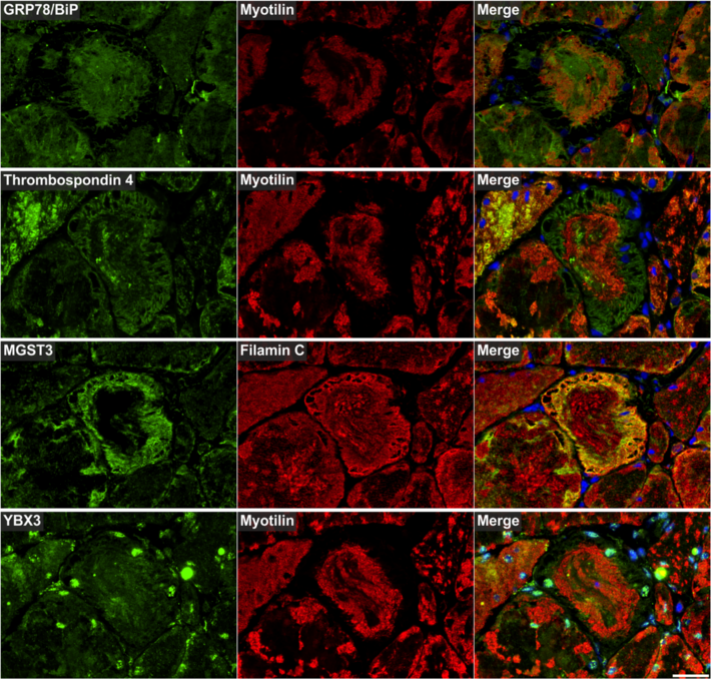
Immunolocalization of proteins involved in unfolded protein response (UPR), oxidative stress response and transcription regulation. Serial skeletal muscle cryosections from a myotilinopathy patient (ID1 in Table 1) were double-stained with antibodies recognizing GRP78/BiP, thrombospondin 4 (both involved in UPR), MGST3 (related to oxidative stress response), and YBX3 (transcription regulation) and either filamin C or myotilin. Nuclei were visualized by DAPI staining. Results of semiquantitative evaluation of signal intensities in the different abnormal areas A1–A3 are specified in Table 3. Scale bar = 50 μm

**Fig. 9:**
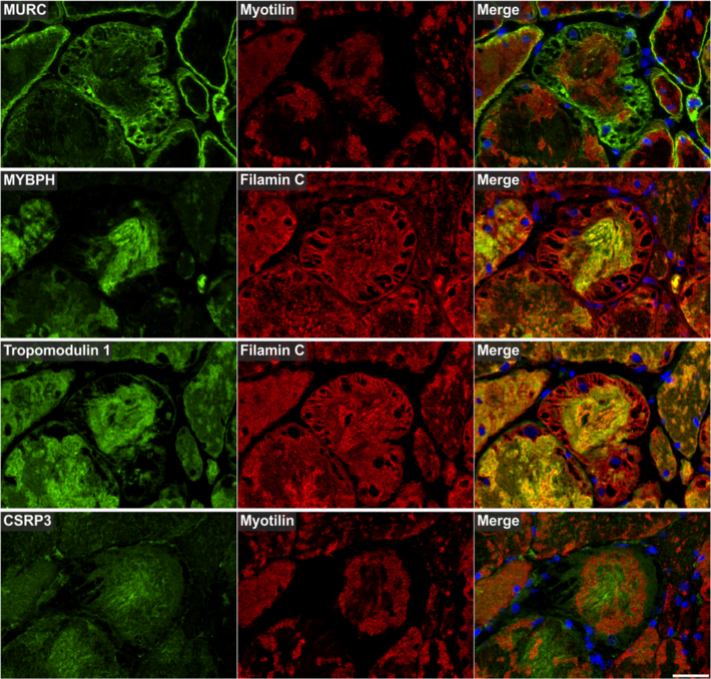
Immunolocalization of proteins involved in actin dynamics. Double immunofluorescence staining was performed on serial skeletal muscle cryosections from a myotilinopathy patient (ID1 in Table 1) by using antibodies directed against MURC, MYBPH, tropomodulin 1, and CSRP3 (a Z-disc protein that is also involved in actin dynamics) and either filamin C or myotilin. Nuclei were visualized by the blue-fluorescent DAPI nucleic acid stain. Data of semiquantitative assessment of immunofluorescence intensities in abnormal areas A1–A3 are listed in Table 3. Scale bar = 50 μm

**Fig. 10:**
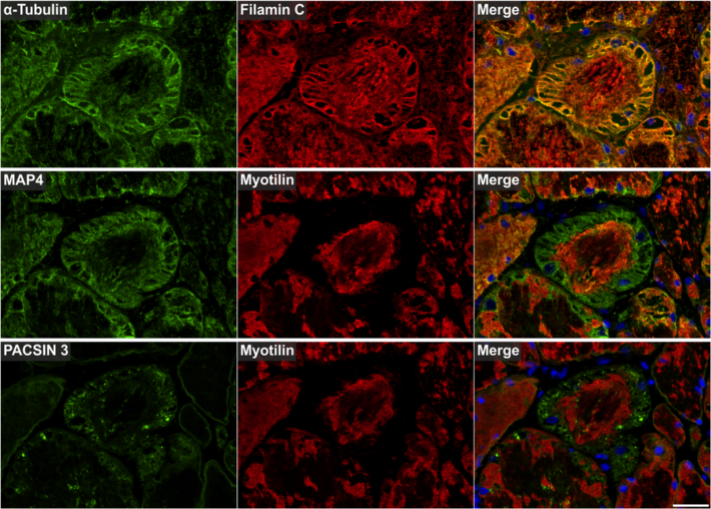
Immunolocalization of proteins involved in microtubule organization and vesicle transport. Serial skeletal muscle cryosections from a myotilinopathy patient (ID1 in Table 1) were double-stained with antibodies recognizing α-Tubulin, MAP4, and PACSIN3 and either filamin C or myotilin. Nuclei were visualized by DAPI staining. Results of semiquantitative evaluation of signal intensities in the different abnormal areas A1–A3 are specified in Table 3. Scale bar = 50 μm

**Fig. 11:**
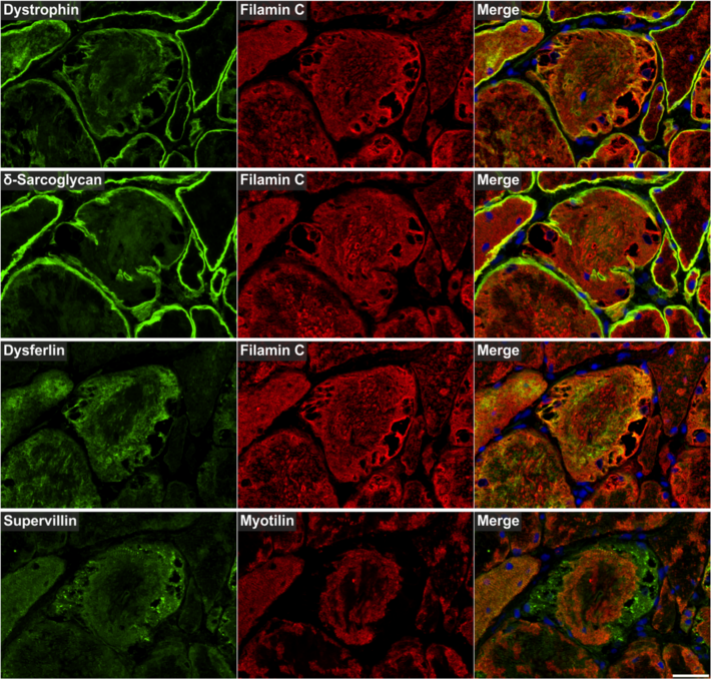
Immunolocalization of sarcolemmal proteins. Double immunofluorescence staining was performed on serial skeletal muscle cryosections from a myotilinopathy patient (ID1 in Table 1) by using antibodies directed against the sarcolemmal proteins dystrophin, dysferlin, supervillain, and δ-sarcoglycan and either filamin C or myotilin. Nuclei were visualized by the blue-fluorescent DAPI nucleic acid stain. Data of semiquantitative assessment of immunofluorescence intensities in abnormal areas A1–A3 are listed in Table 3. Scale bar = 50 μm

**Fig. 12:**
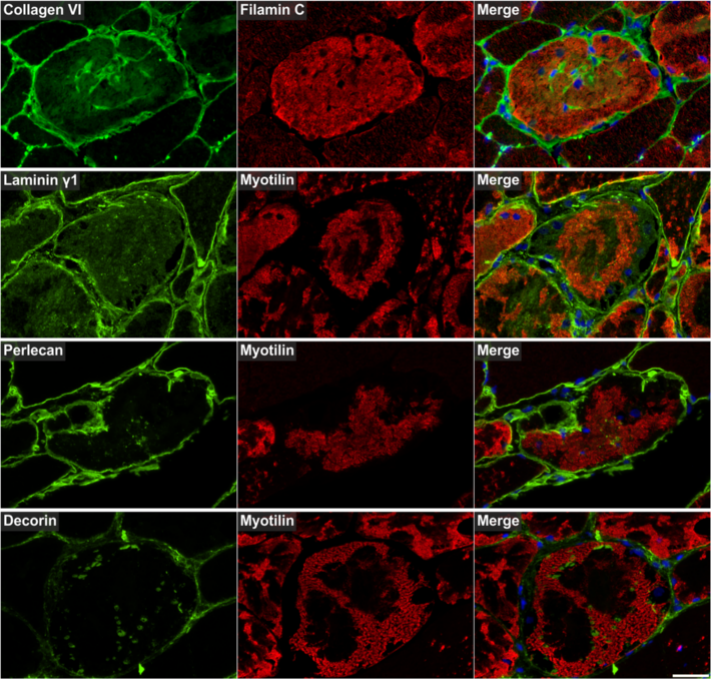
Immunolocalization of extracellular matrix proteins. Serial skeletal muscle cryosections from a myotilinopathy patient (ID1 in Table 1) were double-stained with antibodies recognizing collagen VI, laminin γ1, perlecan, and decorin and either filamin C or myotilin. Nuclei were visualized by DAPI staining. Some abnormal fibers showed an increased immunoreactivity for collagen VI and laminin γ1 in areas of filamin C aggregation and collagen VI also a strong accumulation in some areas with lacking immunoreactivity for filamin C. Immunoreactivities for perlecan and decorin were predominantly increased in areas between and around myotilin-positive aggregates. Scale bar = 50 μm

Myotilin localized in sarcoplasmic aggregates of variable size and shape (Figs. 4, 5, 6, 7, 8, 9, 10 , 11 and 12). The proportion of abnormal fibers with myotilin-positive aggregates varied between <10 % and >50 % in sections of muscle samples from different patients (Additional file 4: Figure S2). Consistent with proteomic analysis, other proteins assessed by immunofluorescence studies also showed an accumulation in at least a part of the myotilin-positive protein deposits (Figs.. 4, 5, 6, 7, 8, 9, 10, 11 and 12). Outside these areas, immunoreactivity for myotilin was usually decreased (Additional file 5: Figure S3) in abnormal fibers, whereas the signal intensities of other evaluated proteins were partly (locally) increased (Figs. 4, 5, 6, 7, 8, 9, 10, 11 and 12). Based on our immunofluorescence findings, we defined three different areas in abnormal fibers (Fig. 4):

Abnormal areas A1: areas with myotilin aggregation (Fig. 4).

Abnormal areas A2: sarcoplasmic areas showing a decreased immunoreactivity for myotilin but a strong accumulation of a subset of Z-disc proteins, chaperones and proteins involved in sarcoplasmic reticulum stress and actin dynamics (Figs. 4, 5, 6, 7, 8, 9, 10 and 11, Table 3).

**Table 3:** Semiquantitative evaluation of signal intensities in immunolocalization studies

Protein	A1	A2	A3
Z-disc (−associated) proteins			
Myotilin	↑↑↑	↓	↓∕-
Filamin C	↑↑∕↑↑↑	↑↑↑	↑↑∕↑↑↑
N-RAP	↑↑	↑↑↑	↑↑
XIRP2	↑↑∕↑↑↑	↑↑↑	↑
Xin	↑↑∕↑↑↑	↑↑↑	(↑)
Obscurin	↑↑	↓	↑↑↑
Myopalladin	↑↑∕↑↑↑	↑↑↑	↑∕↑↑
Desmin	↑↑↑	(↑)	↑↑↑
Plectin	↑	↓	↑↑↑
Nestin	↑∕↑↑	-	↑↑↑
Syncoilin	↑∕↑↑	↑↑↑	↑∕↑↑
Chaperone-assisted selective autophagy (CASA)/NBR1-mediated selective autophagy			
BAG3	↑↑∕↑↑↑	-	↑↑↑
HSPB8/Hsp22	↑↑ ∕ ↑↑↑	↑↑↑	↑↑↑
HSPA8/Hsc70	↑↑	↑	↑↑↑
p62	↑↑↑	(↑)∕↑↑	(↑)
NBR1	↑↑∕↑↑↑	↑	↑↑∕↑↑↑
Further chaperones			
αB-crystallin	↑∕↑↑	↑	↑↑↑
HSPB1/Hsp27	↑∕↑↑	↑↑↑	↑↑↑
Hsp70	↑↑	↑	↑↑↑
Unfolded protein response (UPR) / oxidative stress response/transcription regulation			
BiP	↑↑	↑↑	(↑)
Thrombospondin 4	↑↑∕↑↑↑	↑↑↑	↑↑
MGST3	↑↑↑	-	↑↑↑
YBX3	↑ ∕ ↑↑	↑	↑
Actin dynamics			
MURC	(↑) ∕ ↑↑	(↑)	↑↑↑
MYBPH	(↑) ∕ ↑↑↑	↑↑↑	(↑)
Tropomodulin 1	↑↑ ∕ ↑↑↑	↑↑↑	(↑)
CSRP3	↑	↑↑	↑
Microtubule organization / vesicle transport			
α-tubulin	↑↑	↑	↑↑↑
MAP4	↑↑ ∕ ↑↑↑	(↑)	↑↑↑
PACSIN3	(↑) ∕ ↑↑	-	↑↑↑
Sarcolemmal proteins			
Dystrophin	↑↑	-	↑↑ ∕ ↑↑↑
Dysferlin	↑↑ ∕ ↑↑↑	↑	↑↑ ∕ ↑↑↑
Supervillin	↑ ∕ ↑↑	-	↑↑↑
δ-Sarcoglycan	(↑)	(↑)	(↑)

A1: abnormal area 1, A2: abnormal area 2, A3: abnormal area 3; (↑)∕↑∕↑↑∕↑↑↑: slightly/moderately/distinctly/strongly increased immunoreactivity compared to control fibers; ↓: decreased immunoreactivity compared to control fibers; −: no visible immunoreactivity

Abnormal areas A3: areas surrounding subsarcolemmal vacuoles (Fig. 4) with decreased or absent myotilin immunoreactivity, but strong accumulation of most of the other evaluated proteins (Figs. 4, 5, 6, 7, 8, 9, 10, 11 and 12, Table 3). Intact myofibrils were not observed in A3 areas (Additional file 6: Figure S4)

The results of our extensive comparative immunolocalization studies of i) Z-disc proteins (Figs. 5 and 6), ii) proteins involved in chaperone-assisted selective autophagy (CASA) and NBR1-mediated selective autophagy (Fig. 7), iii) other chaperones and proteins that play a role in unfolded protein response, oxidative stress and transcription regulation (Fig. 8 and Additional file 7: Figure S5), iv) proteins involved in actin dynamics (Fig. 9) and microtubule organization and vesicle transport (Fig. 10), and v) sarcolemmal proteins (Fig. 11) are depicted in the respective figures. Immunofluorescence stainings of control muscle fibers are displayed in Additional file 8: Figure S6. H&E and modified Gomori trichrome stains of muscle fibers presented in Figs. 5, 6, 7, 8, 9, 10 and 11 are shown in Additional file 4: Figure S2. Results of semiquantitative assessment of immunofluorescence signal intensities in areas A1–A3 (compared to normally looking intraindividual control fibers) are listed in Table 3.

Extracellular matrix (ECM) proteins showed an ectopic cytoplasmic expression only in some abnormal fibers (Fig. 12). Collagen VI and laminin displayed a diffuse staining pattern in areas of filamin C aggregation. Collagen VI also strongly accumulated in some areas lacking immunoreactivity for filamin C (Fig. 12). Perlecan and decorin immunoreactivity was especially increased in areas between and around myotilin-positive aggregates (Fig. 12). 14-3-3 γ protein showed an increased immunofluorescence signal predominantly in angulated fibers with filamin C aggregates (Additional file 9: Figure S7). Lamin A/C was located at the nuclear lamina and its over-representation in aggregate samples is obviously caused by centrally located nuclei in aggregate areas (Additional file 7: Figure S5).

## Discussion

Protein aggregation in muscle fibers is the most impressive histological feature of myofibrillar myopathies and seems to play a key role in their pathogenesis. We provide here proteomic data of laser-microdissected sarcoplasmic aggregates from 15 myotilinopathy patients, the largest MFM subtype cohort that has been studied by mass spectrometric analysis so far. The comparison with intraindividual control samples enabled us the identification of proteins which are over-represented in aggregates and therefore appear to be relevant for aggregate formation or modulation. Our innovative approach revealed essential new information about aggregate composition in myotilinopathy that pave the way for future functional studies to investigate disease mechanisms. Furthermore, we identified a novel diagnostic biomarker that can be helpful in differential diagnosis of protein aggregate myopathies.

More than 1000 different proteins were detected by our proteomic analysis of muscle samples from myotilinopathy patients and 72 of them showed a statistically significant over-representation in aggregate samples. Thirty-six of these 72 proteins were further evaluated by immunofluorescence studies on muscle sections from patients. These analyses confirmed and thus validated our mass spectrometric results. In addition, we studied the subcellular distribution of four additional, functionally interesting proteins (GRP78/BiP, CSRP3, δ-sarcoglycan, thrombospondin 4), which were identified as over-represented in aggregate samples but slightly missed the level of statistical significance. We found that abnormal fibers also displayed an increased immunoreactivity for these proteins. Only ten of the in total 76 proteins that were enriched in aggregates were previously described components of deposits in myotilinopathy, namely myotilin [4, 11], desmin [4, 11], filamin C [13, 20], αB-crystallin [4, 11], Xin [16], Xirp2 [16], plectin [11], dystrophin [4, 11], NBR1 [21] and p62 [15]. This implies that 66 aggregate proteins were newly identified. However, considering the large number of identified proteins, false positive results cannot be ruled out. We therefore focus our discussion on findings that we could verify by immunolocalization studies.

We categorized the detected proteins by their main function and found that Z-disc-associated proteins were the most abundant aggregate components. More than 70 % of peptides from over-represented proteins were assigned to this group and the proportion of these Z-disc proteins related to the total number of identified peptides was 3.7 fold higher in aggregate samples compared to controls. Referred to the proportion of specific over-represented proteins in aggregate samples (see [17] for a discussion of limitations of our proteomic approach), myotilin was one of the top proteins. Only desmin and filamin C showed a higher proportion than myotilin. Most of the other Z-disc proteins identified as accumulated in aggregate samples are interaction partners of desmin or filamin C: plectin, syncoilin, and nestin bind to desmin [22–24] and Xin, Xirp2, obscurin, and N-RAP interact with filamin C [17, 25]. Desmin and filamin C are indirectly connected via sarcolemmal proteins [9, 24, 26, 27] and chaperones [17, 28–30] that also showed an over-representation in aggregate samples (see Fig. 2). Our results therefore indicate that protein components of aggregates in myotilinopathy largely belong to a network of interacting proteins. This is in accordance with our findings in filaminopathy [17] and desminopathy [18] and suggests that MFM aggregates are rather structured formations than simple random depositions of aggregate-prone proteins. The finding that Z-disc proteins are the most abundant over-represented proteins in aggregates also corresponds to results of ultrastructural studies indicating that protein aggregation in MFM primarily emanates from Z-discs [4, 5].

The second group of aggregate components consists of sarcolemmal and extracellular matrix proteins. An accumulation of sarcolemmal proteins in aggregate areas has already been described in myotilinopathy and other MFM subtypes including filaminopathy and desminopathy [4, 11, 16, 31, 32]. This may be attributable to protein-protein interactions, e.g. of dystrophin with plectin [33] or between δ-sarcoglycan and filamin C [27]. However, the mechanisms by which extracellular matrix proteins are translocated to sarcoplasmic aggregates remain unclear. Since some of the detected ECM proteins (i.e. collagen VI) are not even expressed in skeletal muscle cells [34], it is tempting to speculate that they are translocated to the sarcoplasm via increased endocytosis. Indeed, we detected an accumulation of proteins in abnormal fibers which are involved in vesicle transport and endocytosis, e.g. PACSIN 3, but the relevance of this finding has to be further investigated.

The third group comprises proteins involved in protein quality control and degradation. A dysfunction of protein degradation mediated by the ubiquitin-proteasome system (UPS) and autophagic pathways seems to play a pivotal role in MFM [13, 15, 35, 36]. In this context, it is an interesting feature of all validated ECM proteins of group 2 that they modulate autophagic signaling pathways via interactions with specific receptors. Decorin, collagen VI and perlecan are activators of autophagy whereas laminin acts as inhibitor (see [37] for review). This demonstrates that several aggregate proteins have various functions and could be assigned to more than one group. The third group includes further proteins with an effect on autophagic protein degradation. The chaperone GRP78/BiP and thrombospondin 4, both detected as over-represented in aggregate samples, are involved in endoplasmic reticulum stress and unfolded protein response. In addition, it has recently been shown that prolonged proteasomal inhibition and cytosolic misfolded proteins induce an N-terminal arginylation of GRP78/BiP [38]. Arginylated GRP78/BiP (R-BiP) in turn associates with misfolded proteins and binds the autophagic adaptor p62. This finally induces a selective lysosomal co-degradation of R-BiP and p62 together with associated cargoes including misfolded proteins [38]. A strong immunoreactivity of myotilinopathy aggregates for p62 has already been reported [15]. Our proteomic analysis now revealed that the proportion of p62 in aggregate samples was 52-fold higher than in controls underlining the extreme extent of p62 enrichment in aggregate areas. An activation of the R-BiP/p62-mediated autophagic degradation pathway may be explained by an impairment of the UPS in myotilinopathy, evidenced by an accumulation of mutant ubiquitin in abnormal fibers [15]. p62 is also involved in chaperone-assisted selective autophagy (CASA), a tension-induced autophagy pathway essential for mechanotransduction in muscle fibers [39, 40]. The CASA complex comprises the chaperones HSPB8/Hsp22 and HSPA8/Hsc70, and the co-chaperone BAG3 that were also over-represented in myotilinopathy aggregates. CASA facilitates degradation of damaged Z-disc proteins including filamin C [39, 40]. We therefore assume that aggregation of Z-disc proteins in myotilinopathy induces an increased expression of CASA components. The finding that abnormal fibers in filaminopathy also show an increased immunoreactivity for CASA proteins [36] suggests that this pathway may be generally important in the pathogenesis of MFM. Another accumulated protein involved in selective autophagosomal protein degradation is NBR1, an autophagic adaptor protein with functions similar to those of p62. Myosin binding protein H (MyBPH) seems to be involved in autophagosomal membrane maturation processes [41], while the chaperones HSPB1/Hsp27 and αB-crystallin play a role in proteasomal protein degradation [42–44].

Taken together, our proteomic analysis identified several proteins that seem to play a role in the complex regulation of protein degradation in myotilinopathy. We suppose that protein aggregation in abnormal muscle fibers induces increased expression of proteins of the third group which interact with or support the degradation of proteins involved in aggregate formation. Since these proteins are potential therapeutic targets, this may be relevant for the development of new treatment strategies. Indeed, it has been shown that e.g. induction of chaperone expression, including proteins that are over-represented in aggregates, efficiently prevented protein aggregation in cell and mouse models of MFM subtypes [45–49]. However, it is unclear if a simple over-expression of proteins involved in degradation processes could be sufficient to eliminate massive protein aggregates. In abnormal fibers impairment of the UPS and autophagy, and factors like oxidative stress may contribute to a resistance of aggregates to degradation [13, 15, 32, 36, 50].

Our immunolocalization studies not only confirmed our proteomic findings but surprisingly also revealed different regions of protein accumulation in abnormal fibers. A1 areas correspond to myotilin-positive aggregates. These areas were collected as aggregate samples by our laser microdissection approach and showed an accumulation of the proteins that were identified as over-represented in aggregate samples by mass spectrometric analysis. Abnormal fibers usually displayed a decreased myotilin immunoreactivity outside area A1. This suggests the assumption that there might be two different kinds of effects of MFM causing *MYOT* mutations: a toxic gain-of-function leading to myotilin-positive protein aggregates and a loss-of-function caused by a shift in subcellular distribution with a deficiency of myotilin at Z-discs that impairs the integrity of myofibrils. A combination of toxic gain- and loss-of-function has already been discussed in other protein aggregation disorders, e.g. in neurodegenerative diseases [51, 52].

The loss-of-function theory in myotilinopathy is supported by identification of abnormal areas A2. These regions showed a decreased myotilin signal but strong accumulation of proteins involved in myofibrillar stabilization, remodeling and repair, such as (co-)chaperones [39, 40, 53–55], proteins with a regulatory role in actin dynamics [41, 56–58], and the Z-disc-associated proteins filamin C, N-RAP, Xin and Xirp2 [25, 54, 59–64]. This may be an attempt of abnormal fibers to protect and repair myofibrils that are disturbed due to local myotilin deficiency. In addition, a lack of further proteins may also be relevant as we detected a decreased immunoreactivity of A2 areas for e.g. the Z-disc-associated proteins obscurin, plectin and nestin. A separate collection and analysis of A2 areas would be a promising, but, from a technical point of view, extremely difficult approach at the moment. A main problem is that no specific marker for these areas has been established so far.

A3 areas are primarily located at the periphery of abnormal fibers and characterized by vacuolar changes, absence of intact myofibrils, and increased immunoreactivity for a plethora of proteins including desmin, plectin, nestin, obscurin, sarcolemmal proteins, and several proteins involved in proteasomal and autophagic pathways. We suppose that proteins actually destined for degradation accumulate in these areas. The formation of large vacuoles may be a consequence of an insufficiency and impairment of autophagy. Interestingly, the immunoreactivity for p62 was only slightly increased in A3 areas, whereas NBR1 strongly accumulated. This suggests that the relevance of distinct selective autophagy pathways differs in A1 and A3 areas. The lack of myotilin in A3 areas indicates that it is trapped in A1 areas. A dynamic flux of other over-represented proteins between different aggregate areas and surrounding sarcoplasm may be possible but this important aspect cannot be assessed by our descriptive immunofluorescence studies. It would be interesting to investigate dynamic processes by e.g. live cell imaging studies but suitable cell culture models of myotilinopathy have not been established so far.

Finally, comparison of data from our proteomic analysis in myotilinopathy with previous findings in filaminopathy and desminopathy [17, 18] showed striking similarities especially in respect of Z-disc-associated proteins. Desmin, filamin C, myotilin, Xirp2, N-RAP, Xin, αB-crystallin and nestin always ranked among the most abundant over-represented proteins indicating a characteristic basic pattern of aggregate composition in MFM. Differences related to the proportion, ratio and order of proteins allowed the identification of subtype-specific proteomic profiles. The ratio of myotilin to filamin C in aggregate samples was identified as a highly sensitive and specific marker for myotilinopathy. In addition, the ratio of filamin C proportions in aggregate and control samples, previously presented as marker to differentiate filaminopathy from desminopathy [18], is also suitable to discern filaminopathy from myotilinopathy. This again demonstrates that proteomic analysis is a valuable tool in the differential diagnosis of MFM. Future studies in other MFM subtypes and further protein aggregate myopathies have to address the issue if other disease entities also display distinct patterns of diagnostic markers.

### Ethical Standards

All procedures performed in studies involving human participants were in accordance with the 1964 Helsinki declaration and its later amendments or comparable ethical standards and with approval of the ethics committee of the Ruhr-University Bochum (#4368-12).

## Conclusions

Our previous work showed that the combination of laser microdissection with proteomic analysis is a highly sensitive method to decipher the composition of protein aggregates in MFM. Our findings in myotilinopathy presented in this work indicate that Z-disc-associated proteins, especially filamin C, desmin and their binding partners, are the most abundant components of the pathological aggregates in muscle fibers of myotilinopathy patients. The network of interactions between several of the accumulated proteins suggests that sarcoplasmic aggregates are structured formations. New information concerning over-represented chaperones and proteins involved in the UPS and autophagic pathways are important for understanding pathophysiological aspects and may be relevant for the development of treatment strategies. Our extensive immunolocalization studies not only confirmed the results of our proteomic analysis but also identified different regions of protein accumulation and a lack of myotilin outside the aggregates in abnormal muscle fibers. This raised the important question whether a combination of toxic gain-of-function and loss-of-function of myotilin is present in myotilinopathy. The identification of the ratio of myotilin to filamin C in aggregates as a highly sensitive and specific diagnostic marker for myotilinopathy underlines that proteomic analysis can be useful in differential diagnosis of myofibrillar myopathies.

### Informed consent

Informed consent was obtained from individual participants included in the study.

## Additional files

Additional file 1:
**Table S1.** Primary antibodies used in double immunofluorescence studies. (PDF 56 kb) Additional file 2:
**Table S2.** Accession number (Swiss-Prot) and names of genes coding for proteins identified as over-represented in aggregate samples in myotilinopathy. (PDF 61 kb) Additional file 3:
**Figure S1.** Composition of protein aggregates collected from different muscles in MFM caused by different *MYOT *mutations. (TIF 1155 kb) Additional file 4:
**Figure S2.** H&E, trichrome and myotilin stain. (TIF 8337 kb) Additional file 5:
**Figure S3.** Immunolocalization of myotilin in abnormal fibers. (TIF 4017 kb) Additional file 6:
**Figure S4.** Immunolocalization of filamin C and myotilin in a longitudinal muscle section. (TIF 1576 kb) Additional file 7:
**Figure S5.** Immunolocalization of additional chaperones. (TIF 5907 kb) Additional file 8:
**Figure S6.** Immunofluorescence analyses of control muscle fibers. (TIF 5332 kb) Additional file 9:
**Figure S7.** Immunolocalization of 14-3-3 γ and lamin A/C. (TIF 3287 kb)
