# Supplementary material for: New insights into the protein aggregation pathology in myotilinopathy by combined proteomic and immunolocalization analyses
Source: Acta Neuropathol Commun. 2016 Feb 3;4:8. doi: 10.1186/s40478-016-0280-0 (PMC4739336; doi:10.1186/s40478-016-0280-0)
Supplement: Additional file 1: — Table S1. Primary antibodies used in double immunofluorescence studies. (PDF 56 kb) [file 40478_2016_280_MOESM1_ESM.pdf]

Supplementary Table 1 Primary antibodies used in double immunofluorescence studies

Antigen	Clone/number	Source	Company/Reference	Dilution
AlphaB-crystallin	clone G2JF	mouse (mAb)	Novocastra/Leica	1/100
BAG family molecular chaperone regulator 3	ab47124	rabbit (pAb)	Abcam	1/2000
Collagen alpha-3(VI) chain	MAB 3303	mouse (mAb)	Chemicon	1/500
Cysteine and glycine-rich protein 3	ab172952	rabbit (mAb)	Abcam	1/200
Desmin	clone D33	mouse (mAb)	DAKO	1/500
Decorin	HPA003315	rabbit (pAb)	Sigma-Aldrich	1/500
Delta-sarcogylcan	clone δSARC/12C1	mouse (mAb)	Novocastra/Leica	1/100
Dysferlin	clone HAM1/7B4	mouse (mAb)	Novocastra/Leica	1/30
Dystrophin	clone D4/6D3	mouse (mAb)	Novocastra/Leica	1/20
Filamin-C	FLNC d16-20	rabbit (pAb)	BioGenes	1/1000
78 kDa glucose-regulated protein	ab21685	rabbit (pAb)	Abcam	1/1000
Heat shock cognate 71 kDa protein	ab2788	mouse (mAb)	Abcam	1/300
Heat shock 70 kDa protein 1	clone 8B11	mouse (mAb)	Novocastra/Leica	1/20
Heat-shock protein beta-1	clone 2B4	mouse (mAb)	Novocastra/Leica	1/100
Heat shock protein beta-8	ab151552	rabbit (mAb)	Abcam	1/150
Lamin-A/C	clone 636	mouse (mAb)	Novocastra/Leica	1/100
Laminin subunit gamma-1	HPA001909	rabbit (pAb)	Sigma-Aldrich	1/100
Microsomal glutathione S-transferase 3	ab110309	mouse (mAb)	Abcam	1/2000
Microtubule-associated protein 4	HPA038150	rabbit (pAb)	Sigma-Aldrich	1/1000
Muscle-related coiled-coil protein	HPA020973	rabbit (pAb)	Sigma-Aldrich	1/1000
Myopalladin	HPA036298	rabbit (pAb)	Sigma-Aldrich	1/1000
Myosin-binding protein H	ab156763	mouse (mAb)	Abcam	1/1000
Myotilin	clone RS034	mouse (mAb)	Novocastra/Leica	1/20
Nebulin-related-anchoring protein (N-RAP)	custom made	rabbit (pAb)	BioGenes	1/500
Nestin	clone 10C2	mouse (mAb)	Abcam	1/500
Next to BRCA1 gene 1 protein	HPA022999	rabbit (pAb)	Sigma-Aldrich	1/100
Obscurin	HPA021186	rabbit (pAb)	Sigma-Aldrich	1/5000
Perlecan	Code No 1030+	rabbit (pAb)	MPI (Martinsried)	1/5000
Plectin	#46	rabbit (pAb)	Andrä et al. 2003	1/200
Protein kinase C and casein kinase substrate in neurons protein 3	HPA039480	rabbit (pAb)	Sigma-Aldrich	1/100
Sequestosome-1 (p62)	ab56416	mouse (mAb)	Abcam	1/1000
Supervillin	HPA020138	rabbit (pAb)	Sigma-Aldrich	1/500
Syncoilin	HPA028311	rabbit (pAb)	Sigma-Aldrich	1/200
Thrombospondin-4	ab176116	rabbit (pAb)	Abcam	1/50
Tropomodulin-1	ab124034	mouse (mAb)	Abcam	1/1000
Tubulin alpha-4A chain	ab110450	mouse (mAb)	Abcam	1/500
Tubulin beta chain	HPA043640	rabbit (pAb)	Sigma-Aldrich	1/400
Xin actin-binding repeat-containing protein 1 (Xin)	clone XR1B	mouse (mAb)	van der Ven et al. 2006	1/5
Xin actin-binding repeat-containing protein 2 (Xirp2)	clone Xirp2	rabbit (pAb)	van der Ven et al.2006	1/500
Y-box-binding protein 3	HPA034838	rabbit (pAb)	Sigma-Aldrich	1/200

mAb: monoclonal antibody; pAb: polyclonal antibody
