# Supplementary material for: New insights into the protein aggregation pathology in myotilinopathy by combined proteomic and immunolocalization analyses
Source: Acta Neuropathol Commun. 2016 Feb 3;4:8. doi: 10.1186/s40478-016-0280-0 (PMC4739336; doi:10.1186/s40478-016-0280-0)
Supplement: Additional file 2: — Table S2. Accession number (Swiss-Prot) and names of genes coding for proteins identified as over-represented in aggregate samples in myotilinopathy. (PDF 61 kb) [file 40478_2016_280_MOESM2_ESM.pdf]

Supplementary Table 2 Accession number (Swiss-Prot) and names of genes coding for proteins identified as over-represented in aggregate samples in myotilinopathy

Accession number	Protein	Gene
P17661	Desmin	*DES*
Q14315	Filamin C	*FLNC*
Q9UBF9	Myotilin	*MYOT*
A4UGR9	Xin actin-binding repeat-containing protein 2 (XIRP2)	*XIRP2*
Q86VF7	Nebulin-related-anchoring protein (N-RAP)	*NRAP*
Q15149	Plectin	*PLEC*
P12111	Collagen alpha-3(VI) chain	*COL6A3*
Q5VST9	Obscurin	*OBSCN*
Q702N8	Xin actin-binding repeat-containing protein 1 (Xin)	*XIRP1*
P02511	αB-crystallin	*CRYAB*
P48681	Nestin	*NES*
P11055	Myosin-3	*MYH3*
P62805	Histone H4	*HIST1H4A*
P12109	Collagen alpha-1(VI) chain	*COL6A1*
Q13203	Myosin-binding protein H	*MYBPH*
P35555	Fibrillin-1	*FBN1*
O75923	Dysferlin	*DYSF*
P02545	Prelamin-A/C	*LMNA*
Q53GG5	PDZ and LIM domain protein 3	*PDLIM3*
P12110	Collagen alpha-2(VI) chain	*COL6A2*
P11142	Heat shock cognate 71 kDa protein	*HSPA8*
P02452	Collagen alpha-1(I) chain	*COL1A1*
Q13501	Sequestosome-1	*SQSTM1*
P08123	Collagen alpha-2(I) chain	*COL1A2*
P68366	Tubulin alpha-4A chain	*TUBA4A*
P04792	Heat shock protein beta-1	*HSPB1*
P98160	Basement membrane-specific heparan sulfate proteoglycan core protein (Perlecan)	*HSPG2*
P11047	Laminin subunit gamma-1	*LAMC1*
Q9UHQ9	NADH-cytochrome b5 reductase 1	*CYB5R1*
P55268	Laminin subunit beta-2	*LAMB2*
Q2TBA0	Kelch-like protein 40	*KLHL40*
P11532	Dystrophin	*DMD*
P08572	Collagen alpha-2(IV) chain	*COL4A2*
P0DMV8	Heat shock 70 kDa protein 1A	*HSPA1A*
P24539	ATP synthase F(0) complex subunit B1, mitochondrial	*ATP5F1*
P07437	Tubulin beta chain	*TUBB*
P07585	Decorin	*DCN*
O95817	BAG family molecular chaperone regulator 3	*BAG3*
P02461	Collagen alpha-1(III) chain	*COL3A1*
O14880	Microsomal glutathione S-transferase 3	*MGST3*
Q5VXT5	Synaptophysin-like protein 2	*SYPL2*
Q7Z406	Myosin-14	*MYH14*
Q5BKX8	Muscle-related coiled-coil protein	*MURC*

P00387	NADH-cytochrome b5 reductase 3	*CYB5R3*
Q86TC9	Myopalladin	*MYPN*
P27816	Microtubule-associated protein 4	*MAP4*
Q14BN4	Sarcolemmal membrane-associated protein	*SLMAP*
Q9UKS6	Protein kinase C and casein kinase substrate in neurons protein 3	*PACSIN3*
Q14596	Next to BRCA1 gene 1 protein	*NBR1*
O95425	Supervillin	*SVIL*
P61981	14-3-3 protein gamma	*YWHAG*
P28289	Tropomodulin-1	*TMOD1*
Q9H7C4	Syncoilin	*SYNC*
P54289	Voltage-dependent calcium channel subunit alpha-2/delta-1	*CACNA2D1*
P07305	Histone H1.0	*H1F0*
Q9UHB9	Signal recognition particle subunit SRP68	*SRP68*
Q92614	Unconventional myosin-XVIIIa	*MYO18A*
P68371	Tubulin beta-4B chain	*TUBB4B*
Q9NQW7	Xaa-Pro aminopeptidase 1	*XPNPEP1*
Q15773	Myeloid leukemia factor 2	*MLF2*
P35580	Myosin-10	*MYH10*
P15502	Elastin	*ELN*
O75323	Protein NipSnap homolog 2	*GBAS*
P30050	60S ribosomal protein L12	*RPL12*
Q9UJY1	Heat shock protein beta-8	*HSPB8*
P07099	Epoxide hydrolase 1	*EPHX1*
P16989	Y-box-binding protein 3	*YBX3*
P35268	60S ribosomal protein L22	*RPL22*
P18124	60S ribosomal protein L7	*RPL7*
Q01955	Collagen alpha-3(IV) chain	*COL4A3*
P13987	CD59 glycoprotein	*CD59*
Q9Y6X0	SET-binding protein	*SETBP1*
P11021	78 kDa glucose-regulated protein	*HSPA5*
P50461	Cysteine and glycine-rich protein 3	*CSRP3*
Q92629	Delta-sarcoglycan	*SGCD*
P35443	Thrombospondin-4	*THBS4*
