# Supplementary figures and images for: New insights into the protein aggregation pathology in myotilinopathy by combined proteomic and immunolocalization analyses
Source: Acta Neuropathol Commun. 2016 Feb 3;4:8. doi: 10.1186/s40478-016-0280-0 (PMC4739336; doi:10.1186/s40478-016-0280-0)

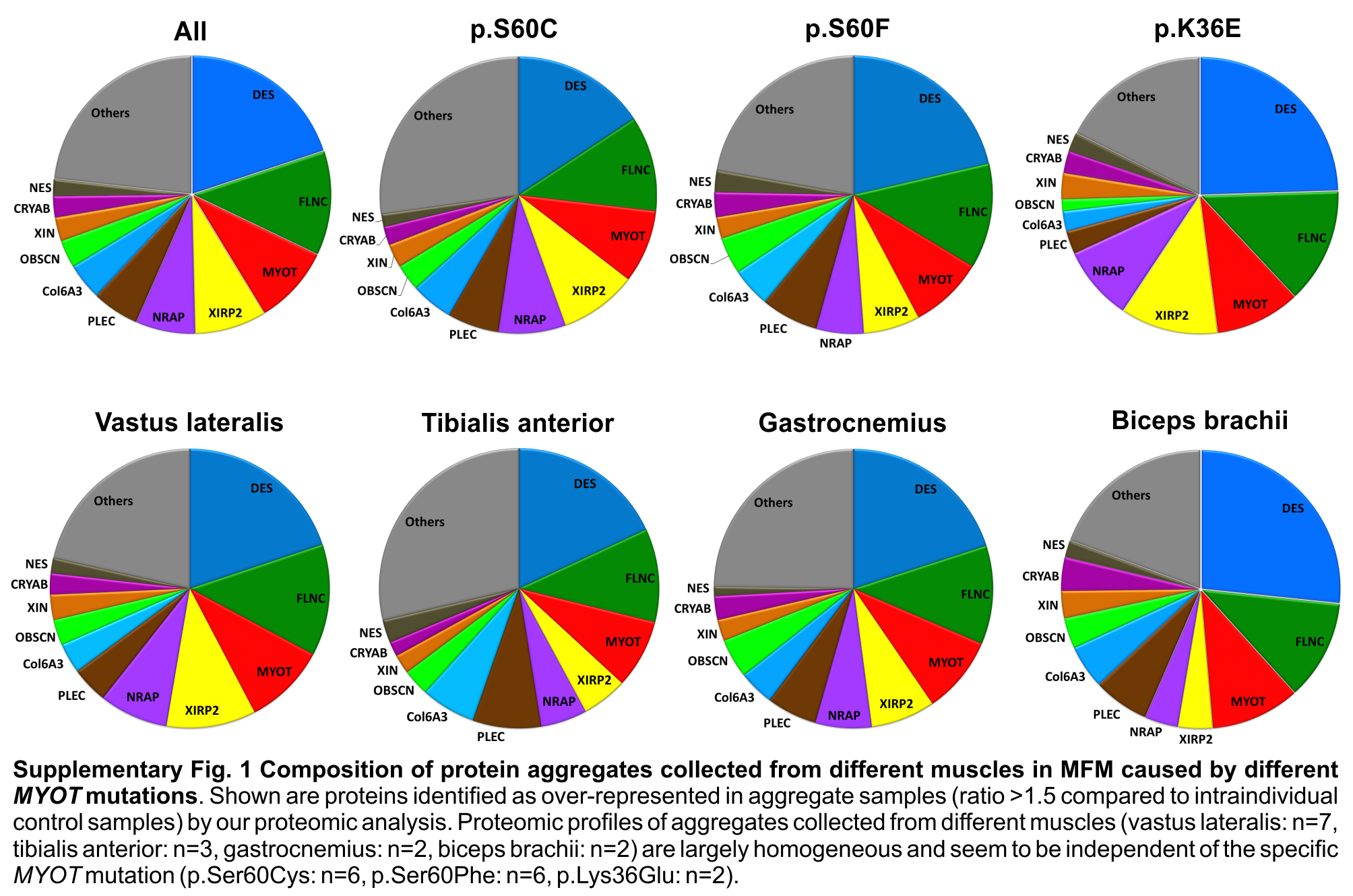

Supplement: Additional file 3: — Figure S1. Composition of protein aggregates collected from different muscles in MFM caused by different MYOT mutations. (TIF 1155 kb) [file 40478_2016_280_MOESM3_ESM.tif]

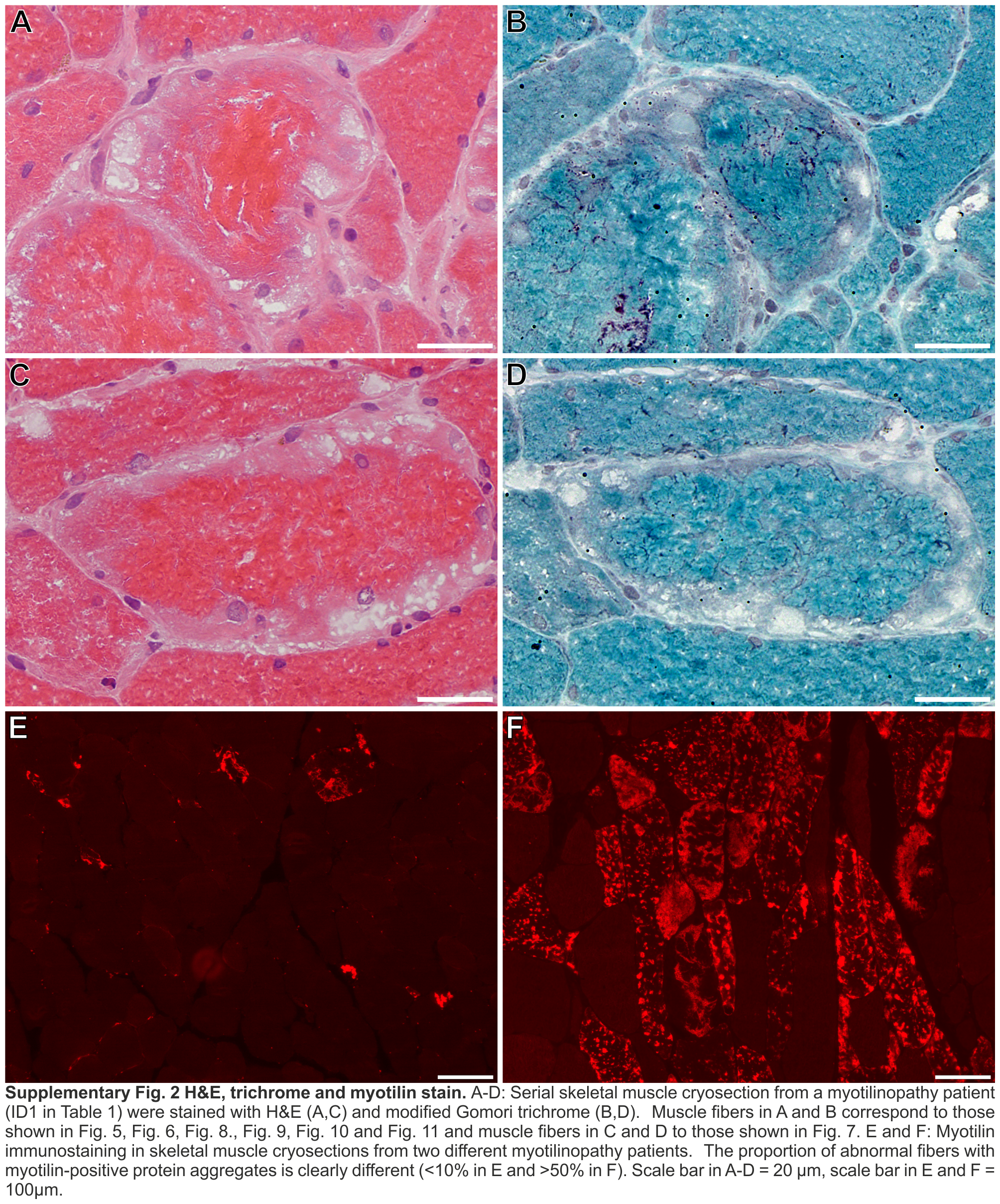

Supplement: Additional file 4: — Figure S2. H&E, trichrome and myotilin stain. (TIF 8337 kb) [file 40478_2016_280_MOESM4_ESM.tif]

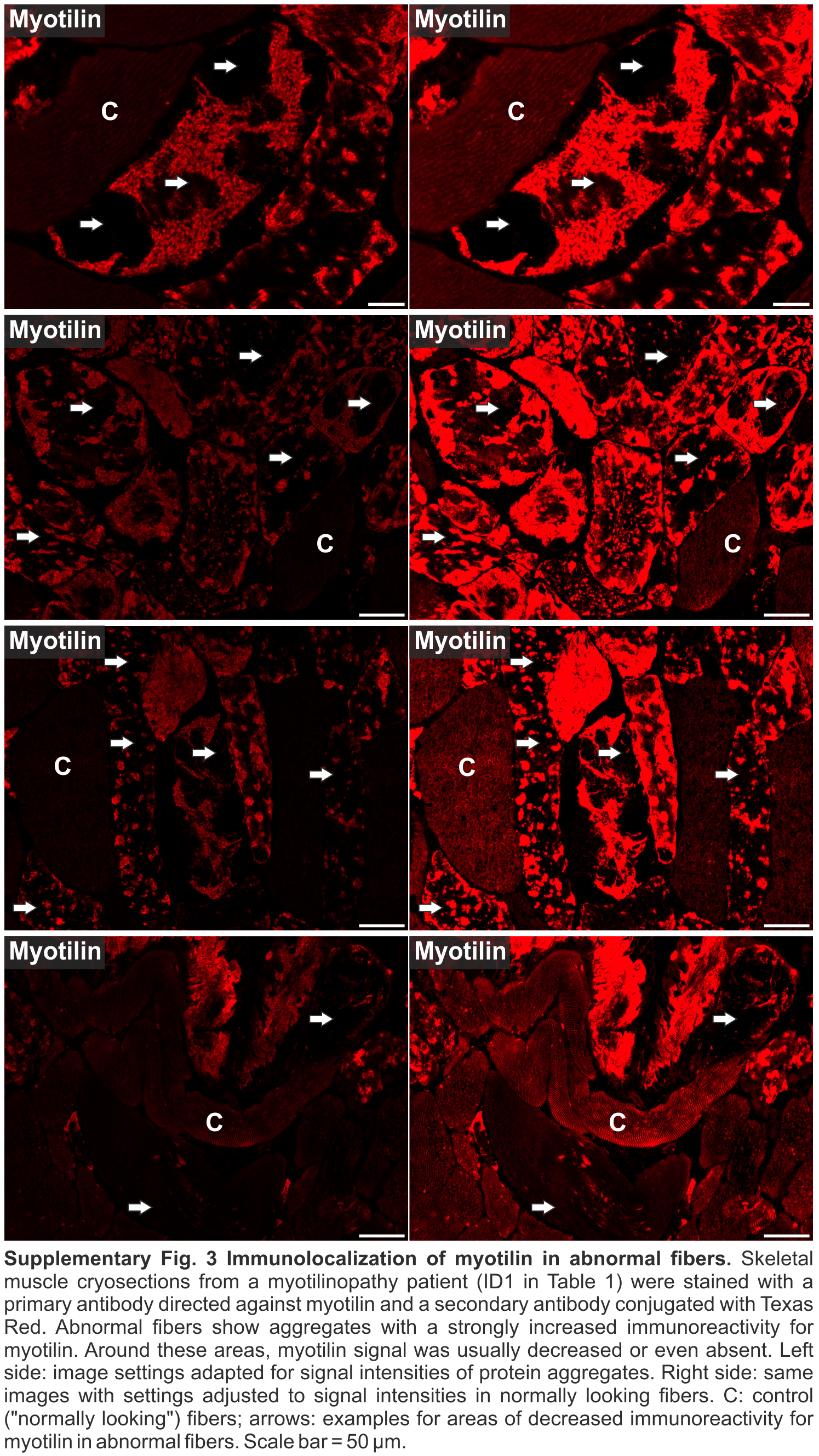

Supplement: Additional file 5: — Figure S3. Immunolocalization of myotilin in abnormal fibers. (TIF 4017 kb) [file 40478_2016_280_MOESM5_ESM.tif]

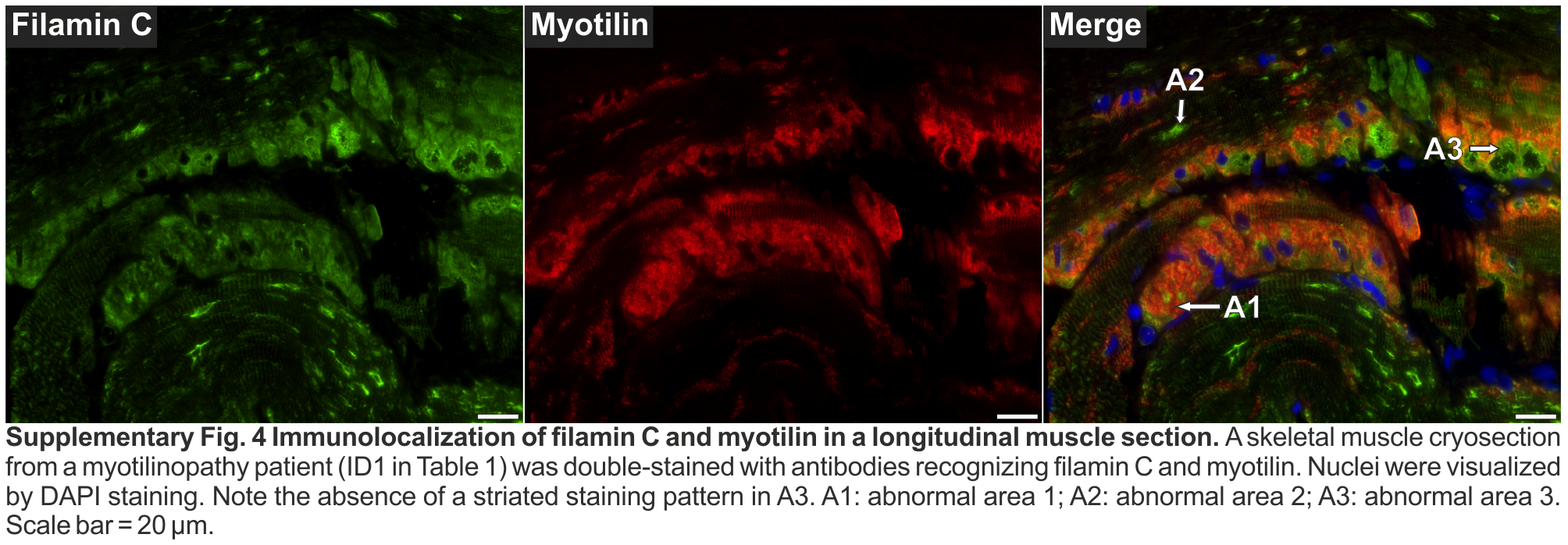

Supplement: Additional file 6: — Figure S4. Immunolocalization of filamin C and myotilin in a longitudinal muscle section. (TIF 1576 kb) [file 40478_2016_280_MOESM6_ESM.tif]

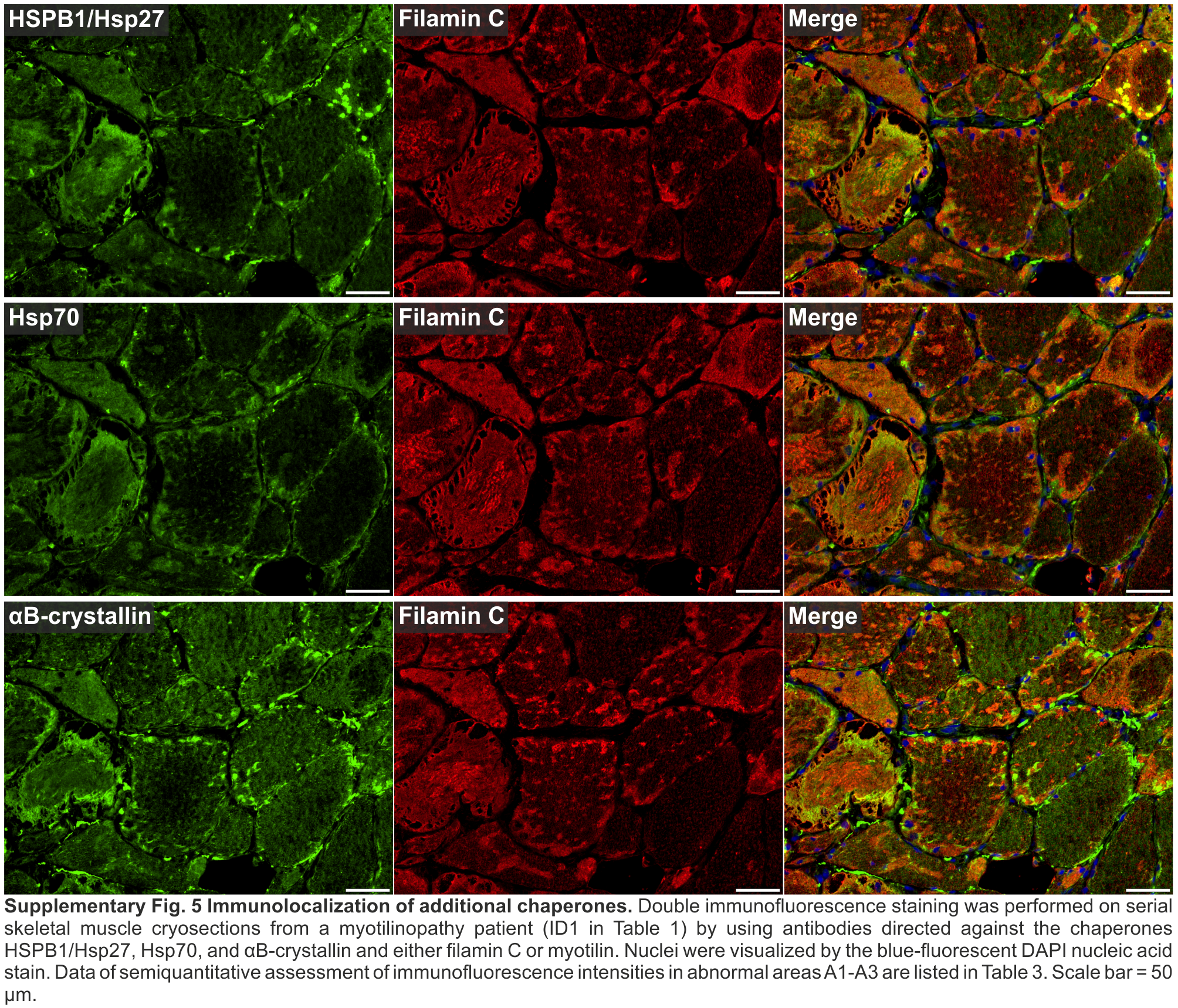

Supplement: Additional file 7: — Figure S5. Immunolocalization of additional chaperones. (TIF 5907 kb) [file 40478_2016_280_MOESM7_ESM.tif]

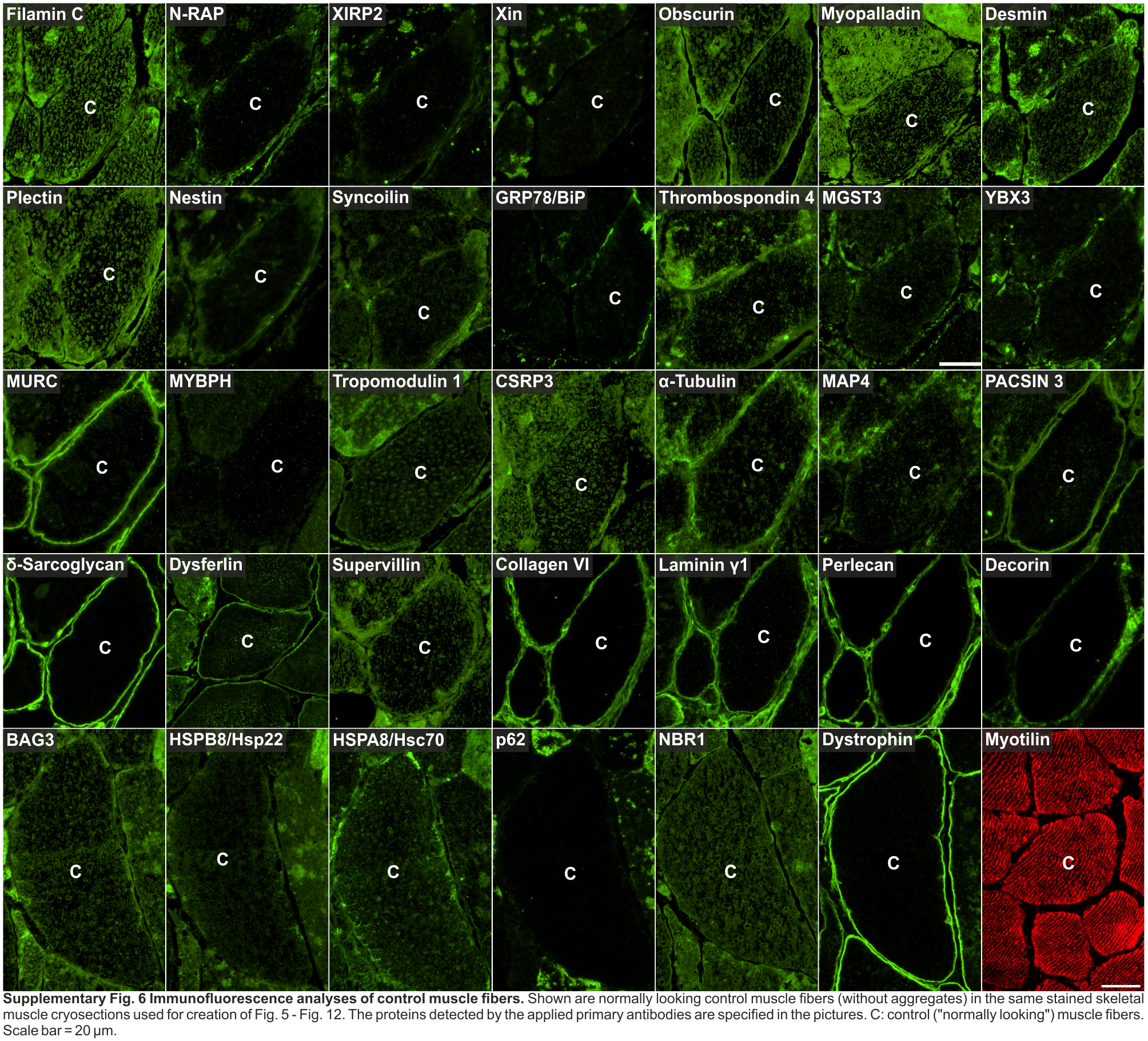

Supplement: Additional file 8: — Figure S6. Immunofluorescence analyses of control muscle fibers. (TIF 5332 kb) [file 40478_2016_280_MOESM8_ESM.tif]

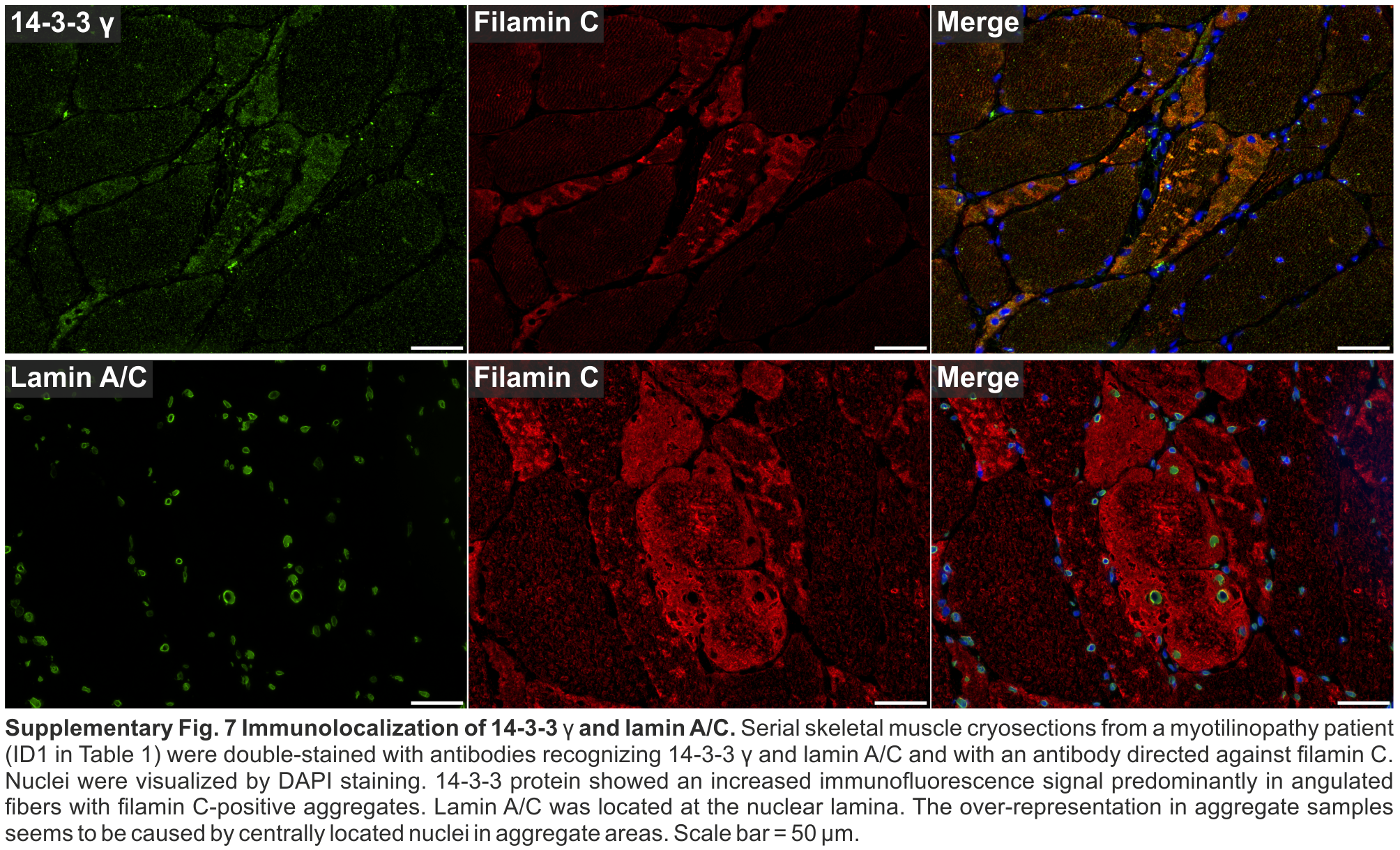

Supplement: Additional file 9: — Figure S7. Immunolocalization of 14-3-3 γ and lamin A/C. (TIF 3287 kb) [file 40478_2016_280_MOESM9_ESM.tif]
